# Bronchial wall parameters on CT in healthy never-smoking, smoking, COPD, and asthma populations: a systematic review and meta-analysis

**DOI:** 10.1007/s00330-022-08600-1

**Published:** 2022-02-22

**Authors:** Ivan Dudurych, Susan Muiser, Niall McVeigh, Huib A.M. Kerstjens, Maarten van den Berge, Marleen de Bruijne, Rozemarijn Vliegenthart

**Affiliations:** 1 Department of Radiology, EB49, University Medical Centre Groningen, University of Groningen, Hanzeplein 1, 9700RB, Groningen, The Netherlands; 2 Department of Pulmonology, University Medical Centre Groningen, University of Groningen, Groningen, The Netherlands; 3 Department of Cardiothoracic Surgery, St. Vincent’s University Hospital, Dublin, Ireland; 4 Department of Radiology and Nuclear Medicine, Biomedical Imaging Group Rotterdam, Erasmus MC, Rotterdam, The Netherlands; 5 Department of Computer Science, University of Copenhagen, Copenhagen, Denmark

**Keywords:** Tomography, X-ray computed, Review, Systematic, Adults, Bronchi, Pulmonary Disease, Chronic Obstructive

## Abstract

**Objective:**

Research on computed tomography (CT) bronchial parameter measurements shows that there are conflicting results on the values for bronchial parameters in the never-smoking, smoking, asthma, and chronic obstructive pulmonary disease (COPD) populations. This review assesses the current CT methods for obtaining bronchial wall parameters and their comparison between populations.

**Methods:**

A systematic review of MEDLINE and Embase was conducted following PRISMA guidelines (last search date 25th October 2021). Methodology data was collected and summarised. Values of percentage wall area (WA%), wall thickness (WT), summary airway measure (Pi10), and luminal area (Ai) were pooled and compared between populations.

**Results:**

A total of 169 articles were included for methodologic review; 66 of these were included for meta-analysis. Most measurements were obtained from multiplanar reconstructions of segmented airways (93 of 169 articles), using various tools and algorithms; third generation airways in the upper and lower lobes were most frequently studied. COPD (12,746) and smoking (15,092) populations were largest across studies and mostly consisted of men (median 64.4%, IQR 61.5 – 66.1%). There were significant differences between populations; the largest WA% was found in COPD (mean SD 62.93 ± 7.41%, *n *= 6,045), and the asthma population had the largest Pi10 (4.03 ± 0.27 mm, *n* = 442). Ai normalised to body surface area (Ai/BSA) (12.46 ± 4 mm^2^, *n* = 134) was largest in the never-smoking population.

**Conclusions:**

Studies on CT-derived bronchial parameter measurements are heterogenous in methodology and population, resulting in challenges to compare outcomes between studies. Significant differences between populations exist for several parameters, most notably in the wall area percentage; however, there is a large overlap in their ranges.

**Key Points:**

• *Diverse methodology in measuring airways contributes to overlap in ranges of bronchial parameters among the never-smoking, smoking, COPD, and asthma populations*.

• *The combined number of never-smoking participants in studies is low, limiting insight into this population and the impact of participant characteristics on bronchial parameters.*

• *Wall area percent of the right upper lobe apical segment is the most studied (87 articles) and differentiates all except smoking vs asthma populations.*

**Supplementary Information:**

The online version contains supplementary material available at 10.1007/s00330-022-08600-1.

## Introduction

Smoking, chronic obstructive pulmonary disease (COPD), and asthma are some of the top non-infective pulmonary health burdens in developed countries [1–3]. Due to an aging population and global smoking rates among others, the number of adults affected with COPD is expected to rise in the future. Both asthma and COPD have a wide variety of phenotypes and presentations, and all have in common the presence of airway inflammation and remodelling [4, 5].

Airway inflammation and remodelling can be measured on CT scans of the thorax. While progress in quantitative CT (QCT) has been made over the past couple of decades, there are many different parameters to evaluate airway disease [6]. Some recent advances have been made in the use of CT-derived bronchial parameters for monitoring disease trajectory, smoking cessation, genetic diversity, and treatment response [7–12]. These demonstrate the potential for quantification and characterisation of a diseased airway.

Current research in this field describes conflicting results for bronchial parameters. Some existing articles describe no differences between groups like lung cancer patients versus healthy individuals, smoking COPD patients versus smoking, and asthma patients versus controls [13–17], while others show significant differences between subgroups, such as COPD GOLD I–IV patients, that would enable further clinical applications like disease monitoring and identification of distinct groups within a population [18–22]. Additionally, some authors report that bronchial parameters vary by sex, age, and other characteristics [23–26], whereas this is not observed by others [27, 28]. To explore this, we conducted a systematic review of bronchial parameter values in never-smoking, smoking, COPD, and asthma populations and compared the resulting pooled values between these populations.

Studies assessing bronchial parameters use a wide range of CT scanning protocols, reconstruction algorithms, and post-processing tools. This may have an impact on radiologic measurements. To enable the possibility of comparing novel research to past studies, we aimed to identify a most used reference technique for bronchial parameter measurement; thus, this review also summarises the current methodologies in use for determining bronchial parameters on CT scans in the never-smoking, smoking, COPD, and asthma populations. We identified previous general reviews on the subject of methodology in bronchial parameter measurement [29]; however, to the best of our knowledge, there are no previous systematic reviews of this subject involving review of the never-smoking population and pooling of never-smoking bronchial parameter data from multiple studies to enable comparison with other populations.

## Methods

This study was conducted following Preferred Reporting Items for Systematic Reviews and Meta-analyses (PRISMA) [30]. The entirety of the screening process was performed using Covidence [31].

### Search strategy

Medline and EMBASE were systematically searched. The last search date was 25/10/2021. The search strings encompassed the key words and index/Mesh terms related to the population: adult, smoking, never-smoking, COPD, asthma, the Intervention: computed tomography scan, and the Outcomes: bronchial wall measurements (e.g. wall measurement, lumen area, wall area etc.). The full search strings are provided in the supplemental material.

### Inclusion/exclusion criteria

The following criteria were required for an article to be included: (1) original empirical research; (2) study population: adults ≥ 18 years old and a focus on at least one of four target populations encompassing common respiratory states: never-smoking or smoking population (without pulmonary disease based on spirometry and GOLD criteria and no history of other pulmonary disease such as pulmonary fibrosis), COPD population, or asthma population; (3) study includes inspiratory chest CT scan for bronchial measurements; (4) research article must be peer-reviewed, English text available.

Exclusion criteria applied were as follows: (1) review article without new experimental data; (2) outlier study population, e.g. coal miners, World Trade Centre firemen; (3) article describing study on phantom/animal/histology specimen only; (4) < 50 participants in the study; (5) non-volumetric CT scan. A scan was considered non-volumetric if the slice increment exceeded slice thickness and was > 2 times larger than voxel size.

The results of the search were processed for eligibility in two steps. Titles and Abstracts were screened by one author for inclusion in full text screening. This was followed by two of three researchers (I.D., S.M., N.McV.) screening the full text for eligibility in the review. Consensus between the two researchers was necessary for inclusion; if consensus could not be reached, the conflict was resolved by the third author. All researchers were blinded to decisions made by one another to reduce bias in the selection process.

Studies included in the methodological systematic review were excluded from the meta-analysis if they had insufficient data for pooling of bronchial parameters.

### Data extraction

Methodologic and study data were collected when available. We focused on tools and methods used in measuring the bronchial walls. These were as follows: reconstruction used for measurement, whether bronchial parameters were normalised to other measurements, e.g. body surface area (BSA), the studied airway branches and generations, and the algorithms and software used for measurement (Figure S1). Following the exclusion of studies with insufficient data for pooling, for each population, we pooled bronchial parameters that were present in two or more of the included studies. These were the following: 3rd generation airway wall area percentage (WA%), wall thickness (WT), luminal area (Ai), Ai normalised to BSA (Ai/BSA), and square root of the wall area of a theoretical airway with an internal perimeter of 10 mm (Pi10) (Figure S2). When multiple articles related to the same bronchial parameters/participants, those articles were grouped by their study name. Per study, data from the article with the largest cohort was used for analysis.

Articles that were eligible for inclusion in pooling of parameters were assessed for bias using a modified Cochrane Risk of Bias tool (RoB 2) [32]. In short, articles were evaluated for Low/High or Some Concerns bias in the domains of Sequence Generation, Allocation Concealment, Incomplete Outcome Data, Selective Outcome Reporting and Other Sources of Bias. A judgement of “High” in any of those domains marks a study as high risk of bias. Irrespective of bias, the reported mean and standard deviation of a bronchial parameter was extracted and included in pooled analysis.

### Statistical analysis

Means and standard deviations from multiple studies were extracted and combined using the Cochrane formula for pooling groups [33]. The resulting pooled values were analysed using one-way ANOVA and Tukey-Kramer HSD post hoc test [34]. An additional meta-analysis of mean differences of COPD vs controls (never-smokers or smokers) for 3rd generation WA% was performed using an inverse-variance with a random effects model, assuming heterogeneity (Deeks and Higgins 2010). To assess for publication bias, a funnel plot was graphed and Eggar’s test performed [35]. A *p* value of < 0.05 was considered statistically significant.

## Results

**Fig. 1:**
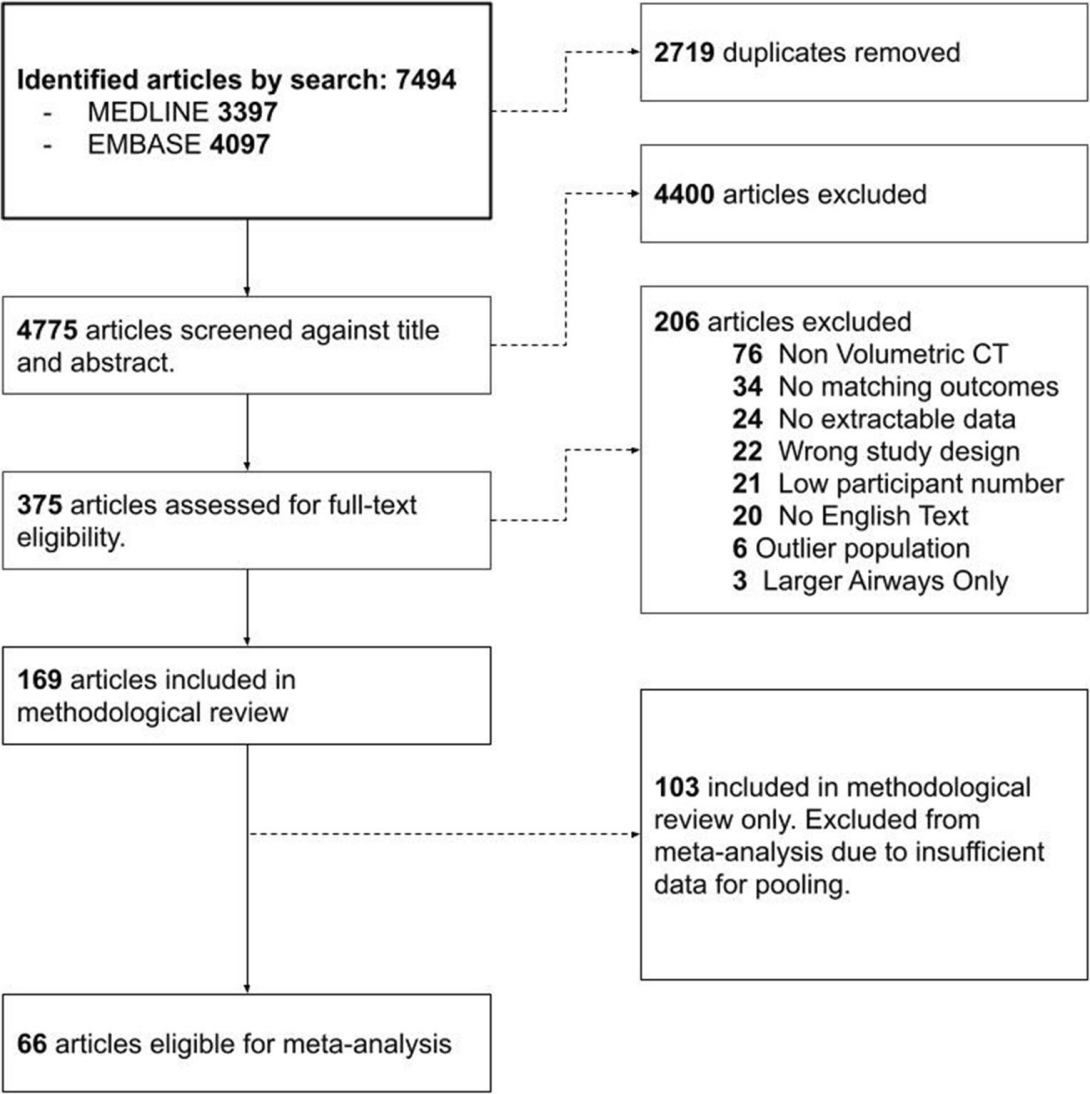
PRISMA flowchart

**Fig. 2:**
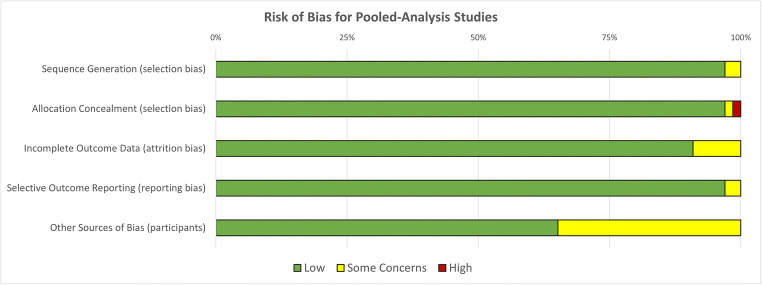
Risk of Bias summary for studies included in pooled analysis (*n*=66)

The search yielded 7,494 articles of which 2,719 were duplicates. Full-text screening was conducted on 375 articles resulting in 169 articles that were included for methodologic evaluation, a summary is provided in supplemental material Table S1. Of these, 66 were eligible for pooling of data, and for comparison of population groups (Figure 1). The most common source of bias was Low, with ”Some Concerns” in the “Other” category due to study participants consisting mostly of men (Figure 2). The details of bias assessment are provided in the supplemental material Table S2.

### Systematic review—population

**Fig. 3:**
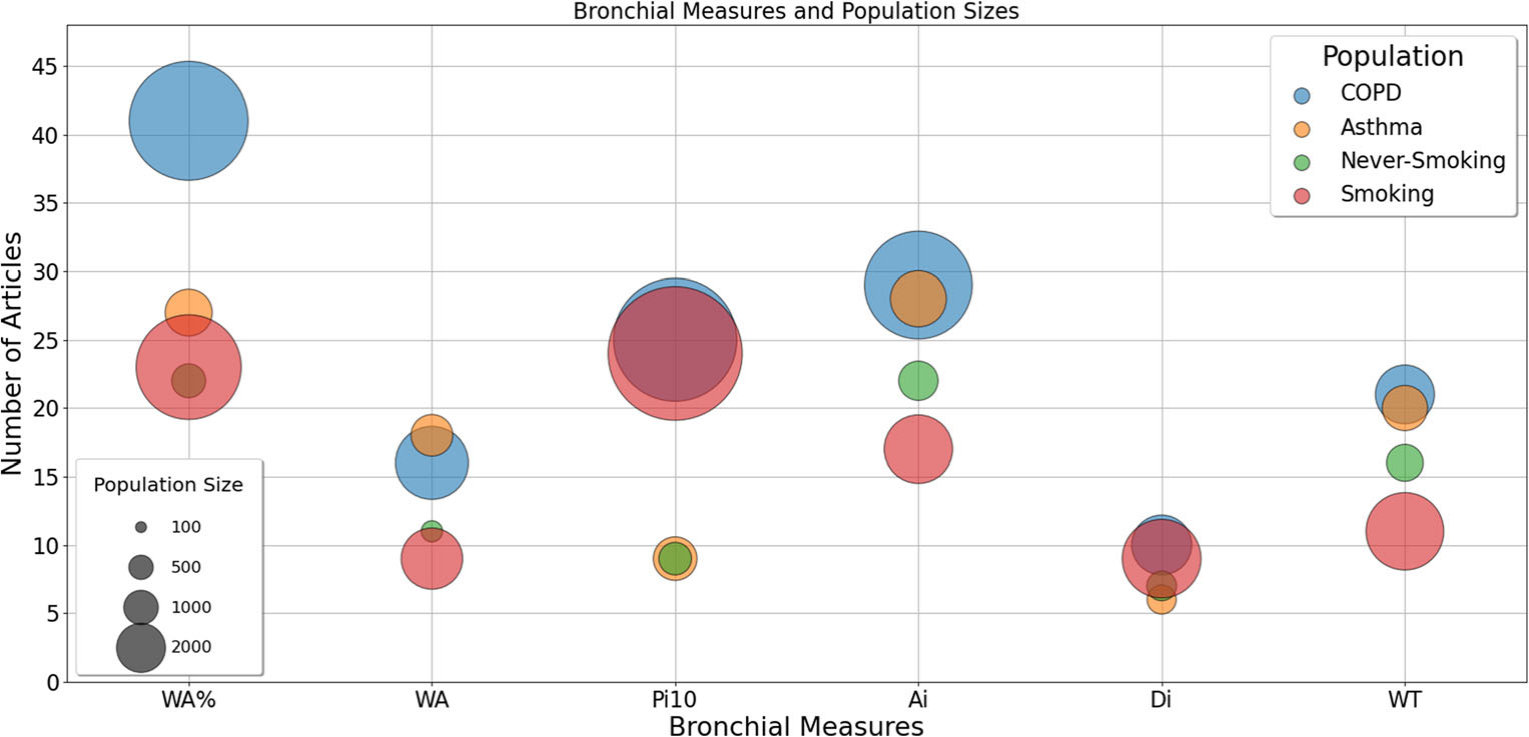
Number of articles investigating a bronchial parameter, with total number of participants per group and across studies indicated by bubble size

We calculated the number of subjects in the four groups and per bronchial parameter measured (Figure 3). Among the reviewed studies, COPD and smoking populations had the largest number of participants: in WA% (*n* = 11,839 COPD and 9,257 smoking) and Pi10 (*n* = 12,746 COPD and 15,092 smoking). Across all measured parameters apart from Di, never-smoking had the lowest numbers of participants. Most of the COPD and smoking participants were men (64.41% male [61.5–66.1%] median [IQR]), while the asthma and never-smoking populations had more women than men (56.44% female [54.7–58.6%]) (Table 1).

**Table 1:** Total number of participants across all studies reporting wall area percentage (WA%), wall area (WA), square root of the wall area of hypothetical airway with internal perimeter of 10mm (Pi10), luminal area (Ai), luminal diameter (Di), and wall thickness (WT). Percentage of participants that are men provided in parentheses. *n*, number of studies

	WA%	WA	Pi10	Ai	Di	WT
COPD	11,839 (65.7) *n * = 41	4,449 (64.9) *n* = 16	12,746 (67.2) *n* = 25	9,731 (62.3) *n* = 29	3,020 (69.1) *n* = 10	2,919 (75.3) *n* = 21
Asthma	1,856 (46.4) *n * = 27	1,463 (40.8) *n* = 18	1,604 (43.2) *n* = 9	2,634 (45.4) *n* = 28	712 (39.6) *n* = 6	1,722 (41.5) *n* = 20
Smoking	9,257 (59.5) *n * = 23	3,168 (60.8) *n* = 9	15,092 (64.4) *n* = 24	3,927 (64.5) *n* = 17	5,207 (61.57) *n* = 9	5,062 (61.3) *n* = 11
Never-smoking	965 (44.9) *n * = 22	378 (43.9) *n* = 11	898 (40) *n* = 9	1,303 (49) *n* = 22	742 (45.3) *n* = 7	1,127 (41.7) *n* = 16

### Methodologic review—image analysis methods

We identified a wide range of methods used to obtain bronchial parameter measurements. Ninety-three of the 169 articles obtained measurements from a reconstructed plane perpendicular to the centreline of the airway, 29/169 articles measured airways cut in cross section on axial slices. 36/169 articles normalised one or more bronchial parameters to body surface area (BSA) or square root of BSA (√BSA).

To determine the airway lumen and wall outline, the full-width half-maximum (FWHM) algorithm was used in 48/169 articles, and graph-cut segmentation was used in 49/169 articles. In 13/169 articles it was unclear which method was used. 43/169 articles used VIDA software, either based on the Apollo or Pulmonary Workstation. Twenty-eight articles used in-house software. The complete summary can be found in Table 2.

**Table 2:** Summary of methodology, indicating the number of articles investigating airway generations or lobes, and the algorithms, methods, and software used for bronchial parameter measurement. *N* = 169 articles. Most studies analysed more than one airway generation. *FWHM*, full-width half maximum

Airway generations		Analysed by lobe		Wall algorithm		Software used	
3	100	RUL	21	Graph-cut	49	In-house	28
4	77	LUL	16	FWHM	48	Apollo VIDA	25
5	68	RLL	14	Intensity-Integration	17	Pulmonary Workstation VIDA	18
6	44	LLL	11	Unclear	13	Airway Inspector 3D Slicer	10
7	22	RML	10	Manual	6	Other	41
8+	17	Lingula	3	Other	8	Unclear	19

### Methodologic review—studied airways and generations

**Fig. 4:**
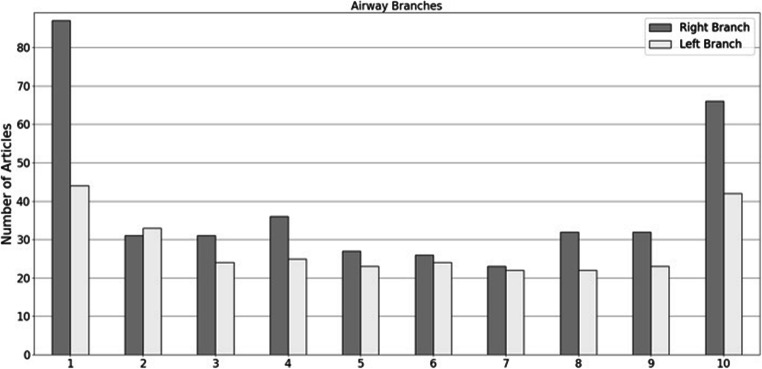
Studied airway branches; grey colour indicates right lung; white colour indicates left lung. Number on *x* axis indicates branch. *y* axis indicates number of articles that include a measure of the specified branch

Of the articles that specified which Boyden Classification [36] airway branches were measured, Right Branch (RB)1 and RB10 were measured in 87/169 and 66/169 articles respectively, and Left Branch (LB)1 ± 2 and LB10 in 77/169 and 42/169 articles (Figure 4). Articles were not included when the airway generation was of a mathematical rather than anatomical distinction, i.e. according to Weibel’s “A” model of the lung [37]. Out of the included articles, the 3rd generation airway was measured in 100/169 articles. 65/169 studies did not provide information on the airway generations that were measured and 4/169 papers measured airways beyond 4th generation and on.

### Pooled analysis—measured bronchial parameters

**Fig. 5:**
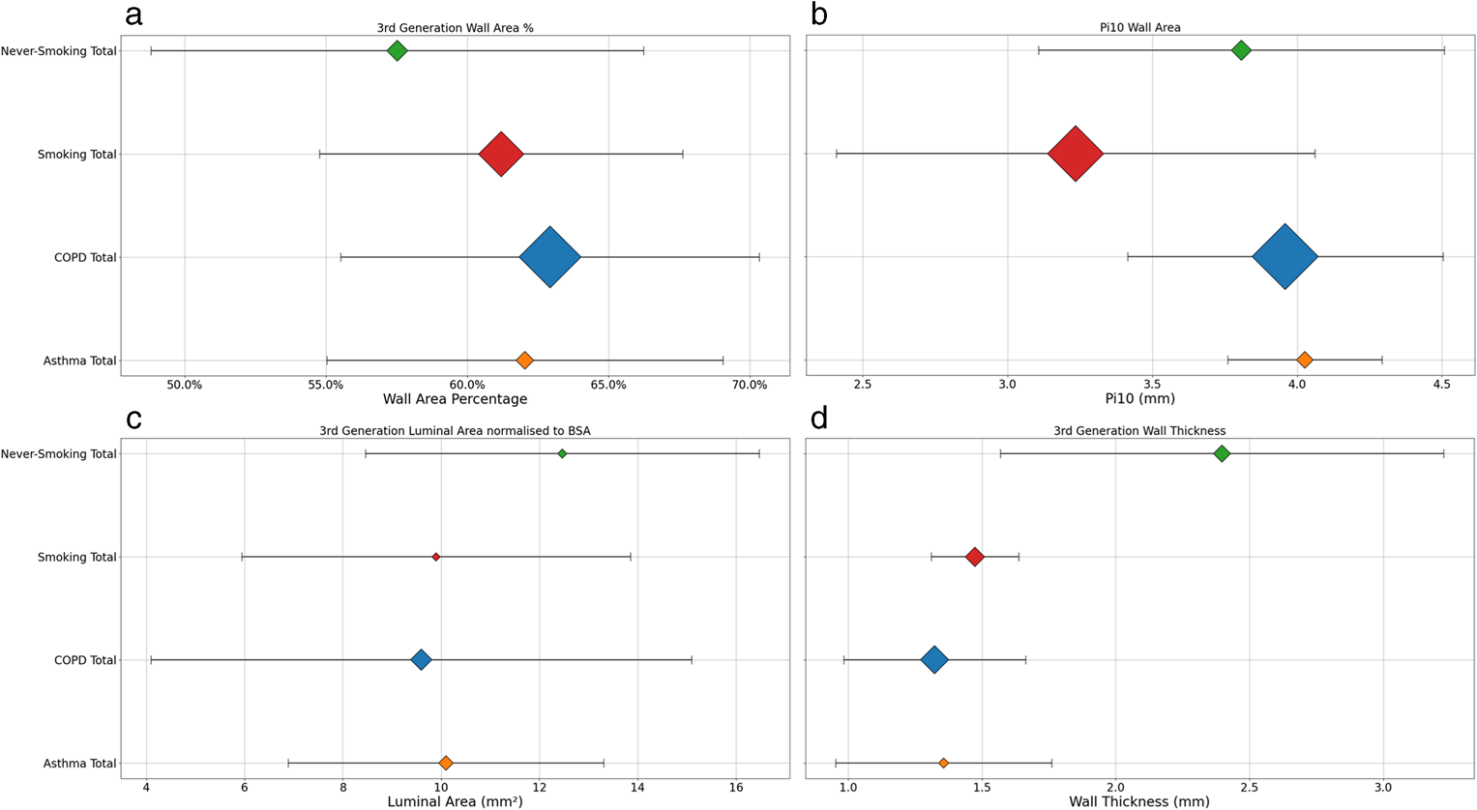
Pooled analysis of (**a**) percentage wall area (WA%) of 3rd generation airways, (**b**) square root of the wall area of hypothetical airway of internal perimeter of 10mm (Pi10), (**c**) luminal area (Ai) normalised to body surface area (BSA), (**d**) wall thickness (WT) of 3rd generation airways. Diamond location is the mean; size indicates relative number of included participants. Error bars are standard deviation

Never-smoking populations had the smallest 3rd generation WA% (57.53 ± 8.71% *n* = 693) followed by smoking populations (61.2 ± 6.43% *n* = 3,228), and asthma populations (62.04 ± 7.0% *n* = 499), with COPD populations having the largest WA% (62.93 ± 7% *n* = 6,045) (Figure 5). One-way ANOVA analysis for WA% showed significant differences between all groups except for smoking versus asthma populations (*p* = 0.07, 95% CI [−0.05%, 1.7%]) (Table 3).

**Table 3:** 95% confidence Interval (in square brackets) and *p* values of one-way ANOVA with Tukey HSD post hoc test comparing the difference between pooled values per population for wall area percentage (WA%), luminal area (Ai), Ai normalised to body surface area (BSA), square root of the wall area of a hypothetical airway with internal perimeter of 10mm (Pi10), and wall thickness (WT)

	Smoking	COPD	Asthma
WA% [%]			
Never-smoking	[2.9, 4.5] *< 0.001*	[4.7, 6.1] *< 0.001*	[3.4, 5.6] *< 0.001*
Smoking		[1.3, 2.1] *< 0.001*	[−0.05, 1.7] *0.07*
COPD			[−0.03, −1.7] *< 0.05*
Pi10 [mm]			
Never-smoking	[−0.5, −0.6] *< 0.001*	[0.1, 0.2] *< 0.001*	[0.1, 0.3] *< 0.001*
Smoking		[0.7, 0.8] *< 0.001*	[0.7, 0.8] *< 0.001*
COPD			[−0.2, 0.02] *0.16*
WT [mm]			
Never-smoking	[−0.9, −1] *< 0.001*	[−1, −1.1] *< 0.001*	[−0.9, −1.1] *< 0.001*
Smoking		[−0.1, −0.2] *< 0.001*	[−0.01, −0.2] *0.02*
COPD			[−0.06, 0.1] *0.8*
Ai [mm^2^]			
Never-smoking	[0.2, 4.6] *0.03*	[−1.9, 2.4] *0.98*	[−5.4, 0.9] *0.26*
Smoking		[−1.8, −3.4] *< 0.001*	[−2.2, −7] *< 0.001*
COPD			[−4.4, 0.4] *0.13*
Ai/BSA [mm^2^]			
Never-smoking	[0.9, 4.1] *< 0.001*	[−1.7, −4] *< 0.001*	[−1.1, −3.6] *< 0.001*
Smoking		[−1.6, 0.9] *0.92*	[−1.1, 1.5] *0.98*
COPD			[−0.3, 1.3] *0.37*

**Fig. 6:**
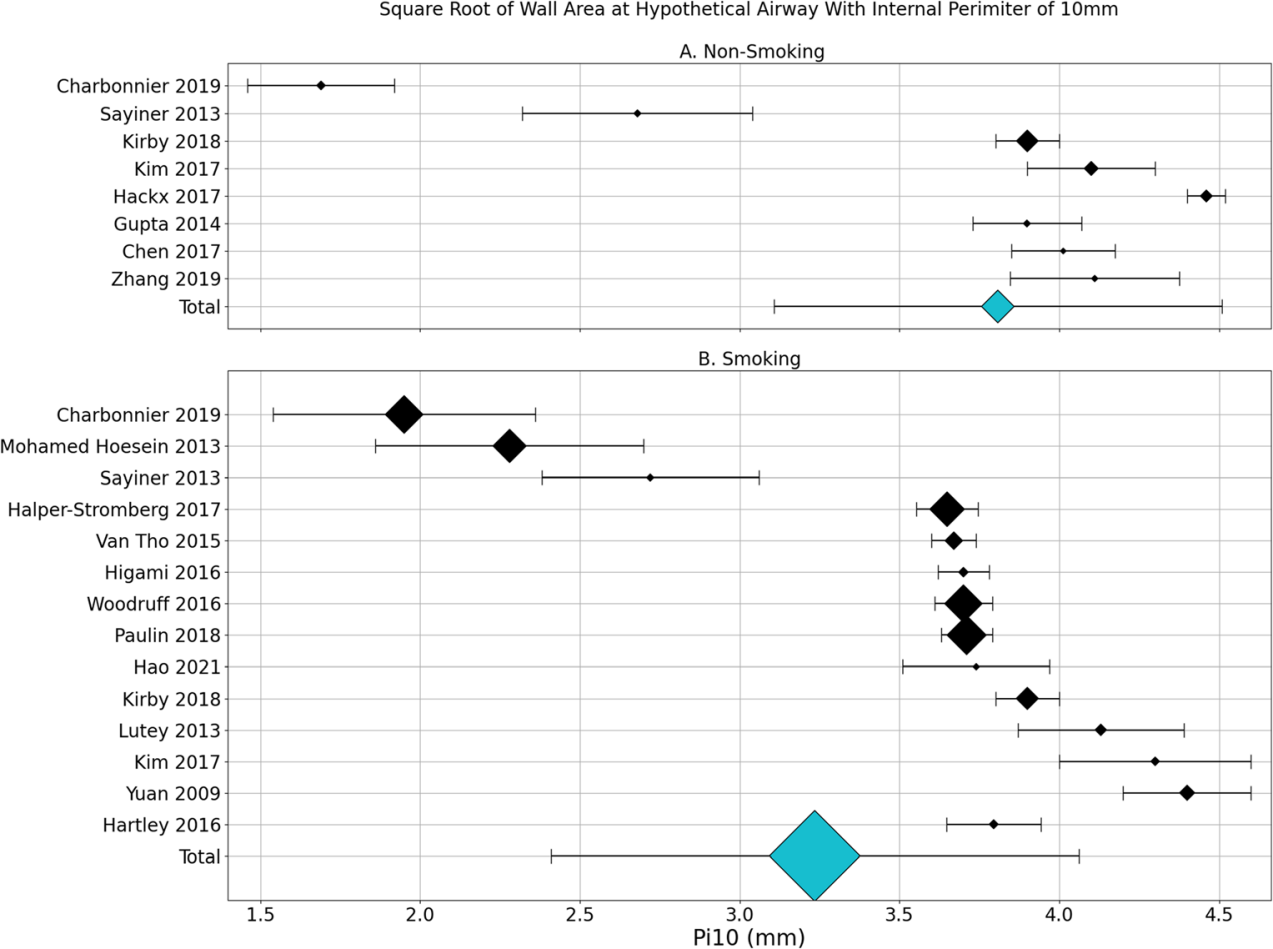
Pooled analysis of square root of wall area of hypothetical airway with internal perimeter of 10mm (Pi10) in never-smoking (A) and Smoking (B) populations. Values are mean ± SD with diamond size indicating relative number of included participants

Pi10 pooled analysis indicates that never-smoking populations have a larger Pi10 (3.81 ± 0.7 mm *n* = 644) than smoking populations (3.23 ± 0.83 mm *n* = 4,942) (*p* < 0.001, 95% CI [−0.5 mm, −0.6 mm]), but smaller than COPD populations (3.96 ± 0.55 mm *n* = 6,887) (*p* < 0.001, 95% CI [0.1 mm, 0.2 mm]) (Figure 6) while asthma populations had the largest Pi10 (4.03 ± 0.27 *n* = 442)(*p* = < 0.001, 95%CI [0.1 mm, 0.3 mm]).

Third generation Ai normalised to BSA was largest in never-smoking populations (12.46 ± 4 mm^2^ *n* = 134), followed by asthma (10.09 ± 3.21mm^2^ *n* = 336), smoking (9.89 ± 3.96mm^2^ *n* = 108), and COPD populations (9.59 ± 5.49mm^2^ *n* = 712). With non-normalised Ai, never-smoking had a smaller Ai (21.69 ± 11.15 mm^2^ *n* = 192) compared to smoking (24.09 ± 12.8 mm^2^ *n* = 2,358), and marginally larger than COPD (21.45 ± 10.58 mm^2^ *n* = 3,323) and asthma (19.45 ± 6.77 mm^2^ *n* = 161).

WT pooled analysis revealed that never-smoking had the thickest 3rd generation walls (2.39 ± 0.83 mm *n* = 460) compared to smoking (1.48 ± 0.16 *n* = 594), COPD (1.32 ± 0.34 *n* = 1,254), and asthma (1.36 ± 0.4 *n* = 163).

### Meta-analysis of 3rd generation WA% for COPD vs controls

Sixteen studies were included in sub analysis of 3rd generation WA%, 6 with never-smokers as controls and 10 with smokers as controls. Overall, 3rd generation WA% was 2.78% larger in COPD compared to controls, *p* ≤ 0.001, 95% CI [1.85, 3.71] (Figure S3). Sub-analysis between COPD and never-smokers shows a difference of 2.59% larger WA% for COPD, 95% CI [1.14, 4.05] and between COPD and smokers WA% was 2.90% larger in COPD, 95% CI [1.71, 4.09]. Egger’s test shows an intercept of 0.35 and *p* = 0.712. The I^2^ ranged from 70.65 to 79.97% in the subgroups, and overall 87.71%.

## Discussion

This systematic review aimed to explore the field of bronchial parameter research in different specified populations. The results show that the study of CT bronchial parameters is biased towards the COPD population’s larger airways. Exploration of airways in never-smokers is needed to solidify knowledge on the differences in bronchial parameters due to participant characteristics. Bronchial walls were most often measured using the full-width half-maximum or the graph-cut method on a plane perpendicular to the centreline of the airway, making full use of the utility of a volumetric CT scan. The 3rd generation right upper lobe apical segment branch was the most often measured bronchial parameter. From a subset of studies, we pooled and compared the reported bronchial parameter values for never-smoking, smoking, asthma, and COPD populations. The wall area percentage of the 3rd generation airway was significantly different between all populations (*p* < 0.001) except between the smoking and asthma populations (*p* = 0.07). The square root of the wall area of a hypothetical airway of 10 mm was significantly larger in the never-smoking compared to smoking population (*p* < 0.001 95% CI [−0.5, −0.6]).

Bronchial parameter research is heavily focused on the COPD and smoking populations. Low numbers of never-smoking participants limit our baseline knowledge of the normal lung parenchyma and bronchial walls as assessed on CT. Our review showed that articles reporting on never-smoking and asthma populations tended to normalise parameters, while articles investigating smoking and COPD did not. Normalisation seeks to control for patient characteristics that affect bronchial wall parameters. The majority of normalisation is performed with body surface area or square root of body surface area due to the similarity of units [38]. Alternative methods of normalisation, such as normalisation to tracheal parameters, have been examined but may require further research to assess robustness [39–41]. Inclusion of more never-smokers in studies may allow for clearer understanding of the interplay between bronchial parameters and participant characteristics such as sex, height, and age, without the confounding factors of smoking and other disease states.

One of the challenges in conducting research in the field of quantitative CT bronchial parameters is determining the optimal CT methodology for bronchial wall measurement. Scanner model and protocol significantly influence the measurements [42, 43], along with participant inspiration levels during the scan [44, 45]. CT scanning is continually advancing, and much of the early research has been focused on individual slices where the airway is cut in cross section according to anatomical properties, e.g. the right upper lobe apical segment airway being almost perpendicular in the axial plane. However, volumetric scanning is increasingly more common and allows for segmentation of the airway tree, in turn allowing for more accurate measurement of the walls. Most articles using volumetric CT scanning employed multiplanar reconstruction when measuring airways, a method that unlocks more bronchial branches for measurement. Despite this, we identified that the larger airways in the upper and lower lobes of the lungs were most often studied; relying on a single location may not adequately capture the complex structural changes that the lungs undergo in disease (e.g. upper vs lower airways [46]). Access to cheap computing allows more complex segmentation and wall measuring tools; however, most articles use FWHM which has been shown by Gierada et. al and Washko et al. to over-estimate the wall thickness [15, 47].

WA% was by far the most measured parameter within all populations and 3rd generation WA% was significantly different between all except smoking versus asthma populations. The meta-analysis focusing on the COPD population vs controls supports the results of the pooled analysis, showing significantly increased WA% in the COPD population. Egger’s test and the funnel plot demonstrate no strong evidence for publication bias for this bronchial parameter. The analysis displayed heterogeneity which was not resolved when the sub-groups were analysed; this indicates that the heterogeneity does not stem from a difference in the populations. Overall 3rd generation WA% appears to be a robust parameter when used to differentiate COPD subjects to controls, despite considerable heterogeneity in the data which may stem from differences in methodology.

Pi10 was distinctly explored in COPD and smoking populations, and less so in asthma and the never-smoking populations. Pooled analysis of bronchial parameters shows significant differences between populations despite different measurement methodologies however with a considerable overlap between the ranges of populations. Pooled Ai normalised to BSA had a smaller range than non-normalised Ai and in both cases the numbers in pooled analyses were low. This may indicate that direct measures are not specific enough to discern between groups, as other participant/pathologic processes play a role in Ai, for example height and sex. Direct measures of bronchial parameters are important building blocks; however, derived markers are more likely to be robust as they correct for confounding factors.

We noted that the pooled values of Pi10 and WT were larger in the never-smoking population compared to smokers, and smaller compared to COPD participants, due to differences of Pi10 measurements in some of the larger studies compared to the others. This was an unexpected finding as current literature indicates that never-smoking individuals have less airway inflammation than smoking, COPD, and asthma populations. The high Pi10 measurement in some studies may be due to several factors. First, Pi10 is calculated by plotting a regression line based on several airway measurements; the location and method of measurements may strongly influence the slope and intercept, leading to differing results [48, 49]. Second, there were more Asian participants in the never-smoking pooled value of Pi10. Ethnicity may play a role and differences between Asian and Caucasian populations have been demonstrated in previous studies [50]. Thirdly, the smoking and COPD populations were predominantly older men with a larger number of participants, while never-smoking populations tended to be younger and included more women. As previous studies have shown, these characteristics play a role in bronchial parameters [51–55]. We were not able to identify suitable measurements to include in the pooled analysis for never-smoking from all studies; however, COPDGene noted a Pi10 of 1.69 ± 0.23 mm in 44 never-smoking individuals [56], which is much lower than the pooled analysis total. This suggests that while Pi10 may be consistent within a study, differences in the methods used to calculate it may not allow for confident comparison between studies.

### Limitations

This study had several limitations. First, the pooled analysis could only include reported means and standard deviations, which assumes a normal distribution in the populations but may not reflect the true distribution. Second, due to the lack of a detailed breakdown of participants in most reviewed literature, it was not possible to perform pooled analysis of sub-groups, and so the pooled values include both men and women, and a wide range of ages, disease states (e.g. non-severe and severe asthma, or GOLD I-IV COPD), and backgrounds (Caucasian, Asian, African-American). Finally, while there are multiple novel potential bronchial parameters emerging due to advancing computation and automation, such as airway tapering and total airway count [57–61], we were able to focus only on the parameters that were available for data extraction. Lastly, of the papers included for meta-analysis, only one obtained post-bronchodilation CT measurements [57]. While post-bronchodilator pulmonary function testing was the norm for studies utilising this technique, it was not used during the CT scan, indicating a difference between the acquisition of spirometry and the CT.

### Conclusions

There are significant differences in bronchial parameters between populations, most notably in the wall area percentage of the 3rd generation airway; however, there is a large overlap in their ranges. While previous studies demonstrate that Pi10 can differentiate disease states within a study, our analysis indicates it may not be a robust parameter when comparing different studies. A paucity of never-smoking participants, along with heterogenous wall measurement methodology, may explain the diverging results from studies on the influence of participant characteristics in bronchial parameters.

## Supplementary information

ESM 1 Examples of wall measurement algorithms. A) Full-Width Half-Maximum (FWHM) measures the Hounsfield Unit intensity along a ray originating from the centre of the lumen and crossing the airway wall. It measures the width of the airway to be the distance between the half of maximum intensity on either side of the peak. B) Graph-Cut. A graph model representation of the airway is cut at the boundary of the lumen and the wall to provide a lumen segmentation and a wall segmentation of the airways. C) Intensity-integration. A method that utilises thresholding to segment the airway wall from the scan and to measure the resulting segmentation wall thickness. D) Multiplanar Reconstruction of the airway. The volumetric scan is reconstructed to straighten out an airway branch, and a measurement is taken perpendicular to the centreline of the airway (in yellow). (PNG 1351 kb)

ESM 2 Bronchial Parameters. A) Wall Thickness (WT), Luminal Area (Ai) in orange, Wall Area (WA) in blue, Wall Area Percentage (WA%). B) Square root of the wall area (√WA) of a hypothetical airway with an internal perimeter (Pi) of 10 mm. The measurements are plotted on a graph and a line of best fit is calculated. The intersection of Pi at 10 mm and √WA is the Pi10 value. The value of the intersect and the slope of the line has an influence on the calculated value of Pi10. (PNG 287 kb)

ESM 3 Summary results for meta-analysis of COPD population 3^rd^ generation Wall Area Percentage (WA%) vs controls. A) Forest plot of mean difference in WA% for individual studies. B) Forest plot of studies included in meta-analysis. COPD-NS-Total = COPD vs Never-Smokers mean difference. COPD-S-Total = COPD vs Smokers mean difference. Q = chi-squared. (PNG 304 kb)

ESM 4 (DOCX 30 kb)

ESM 5 (DOCX 41 kb)

ESM 6 (DOCX 30 kb)

## Abbreviations

Ai: Luminal area
BSA: Body surface area
COPD: Chronic obstructive pulmonary disease
CT: Computed tomography
FWHM: Full-width half-maximum
Pi10: Square root of the wall area of a hypothetical airway with internal perimeter of 10 mm
QCT: Quantitative computed tomography
WA%: Wall area percentage
WT: Wall thickness

## Declarations

### Guarantor

The scientific guarantor of this publication is Prof. Rozemarijn Vliegenthart.

### Statistics and biometry

One of the authors has significant statistical expertise.

### Methodology

• Systematic review

• Meta-analysis
