# Supplementary figures and images for: Bronchial wall parameters on CT in healthy never-smoking, smoking, COPD, and asthma populations: a systematic review and meta-analysis
Source: Eur Radiol. 2022 Feb 22;32(8):5308–18. doi: 10.1007/s00330-022-08600-1 (PMC9279249; doi:10.1007/s00330-022-08600-1)

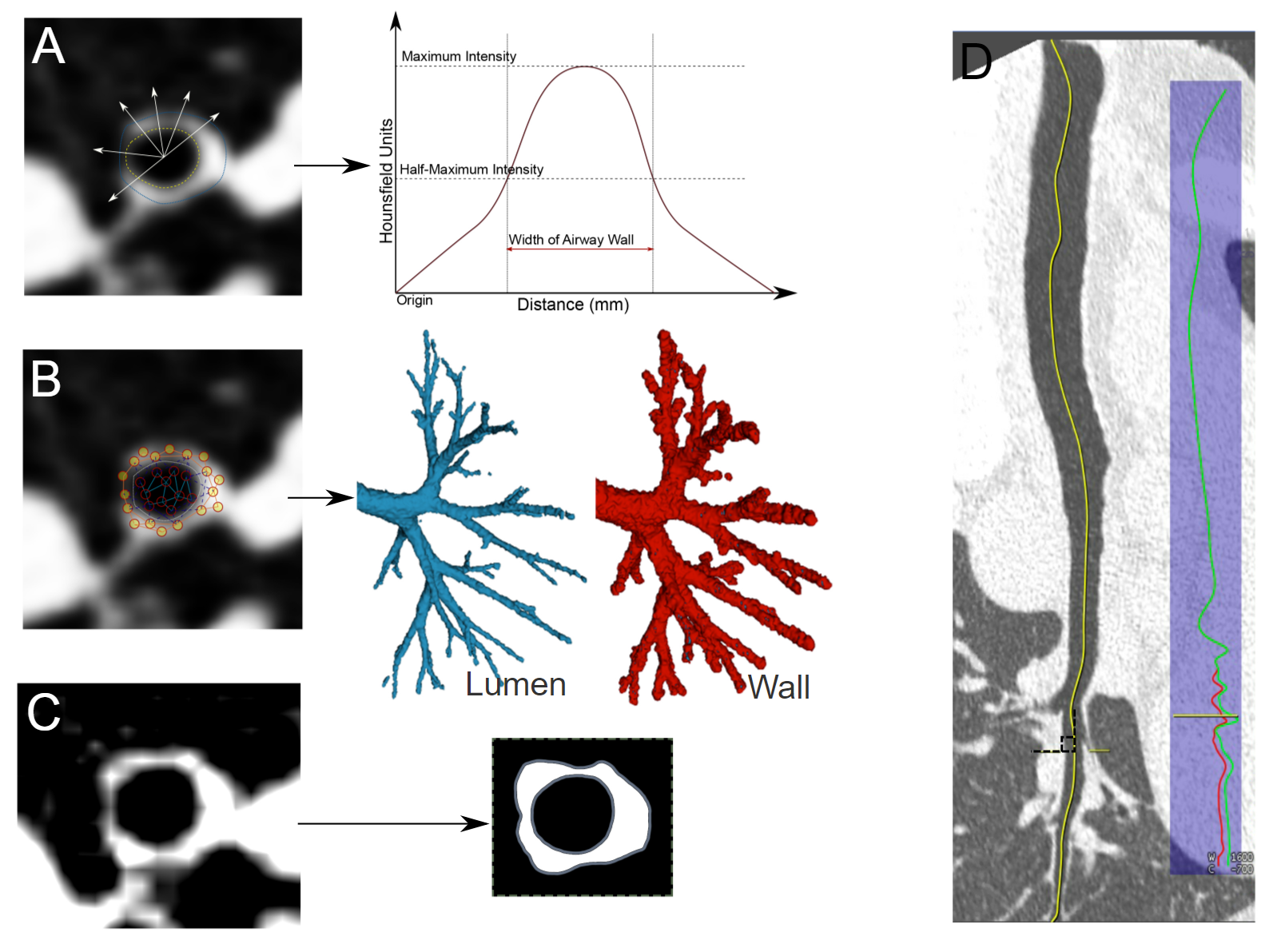

Supplement: Supplementary file 1 — Examples of wall measurement algorithms. A) Full-Width Half-Maximum (FWHM) measures the Hounsfield Unit intensity along a ray originating from the centre of the lumen and crossing the airway wall. It measures the width of the airway to be the distance between the half of maximum intensity on either side of the peak. B) Graph-Cut. A graph model representation of the airway is cut at the boundary of the lumen and the wall to provide a lumen segmentation and a wall segmentation of the airways. C) Intensity-integration. A method that utilises thresholding to segment the airway wall from the scan and to measure the resulting segmentation wall thickness. D) Multiplanar Reconstruction of the airway. The volumetric scan is reconstructed to straighten out an airway branch, and a measurement is taken perpendicular to the centreline of the airway (in yellow). (PNG 1351 kb) [file 330_2022_8600_MOESM1_ESM.png]

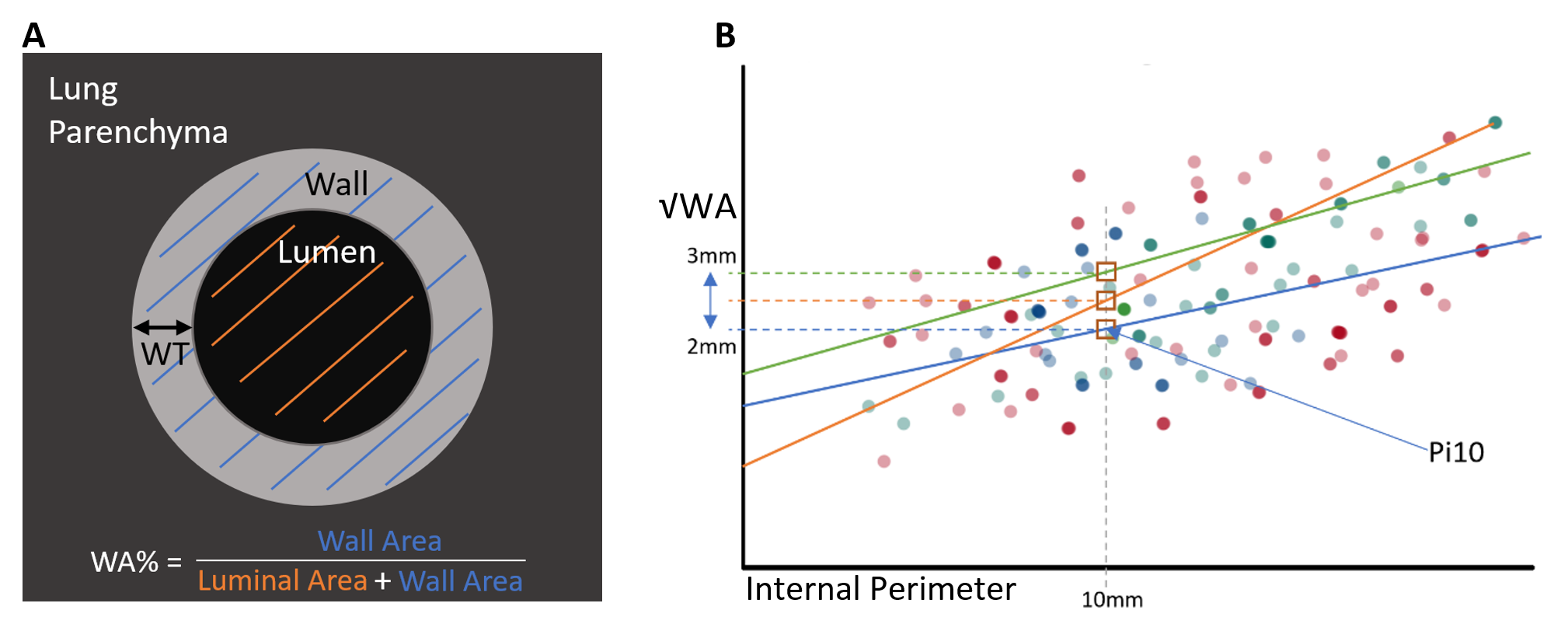

Supplement: Supplementary file 2 — Bronchial Parameters. A) Wall Thickness (WT), Luminal Area (Ai) in orange, Wall Area (WA) in blue, Wall Area Percentage (WA%). B) Square root of the wall area (√WA) of a hypothetical airway with an internal perimeter (Pi) of 10 mm. The measurements are plotted on a graph and a line of best fit is calculated. The intersection of Pi at 10 mm and √WA is the Pi10 value. The value of the intersect and the slope of the line has an influence on the calculated value of Pi10. (PNG 287 kb) [file 330_2022_8600_MOESM2_ESM.png]

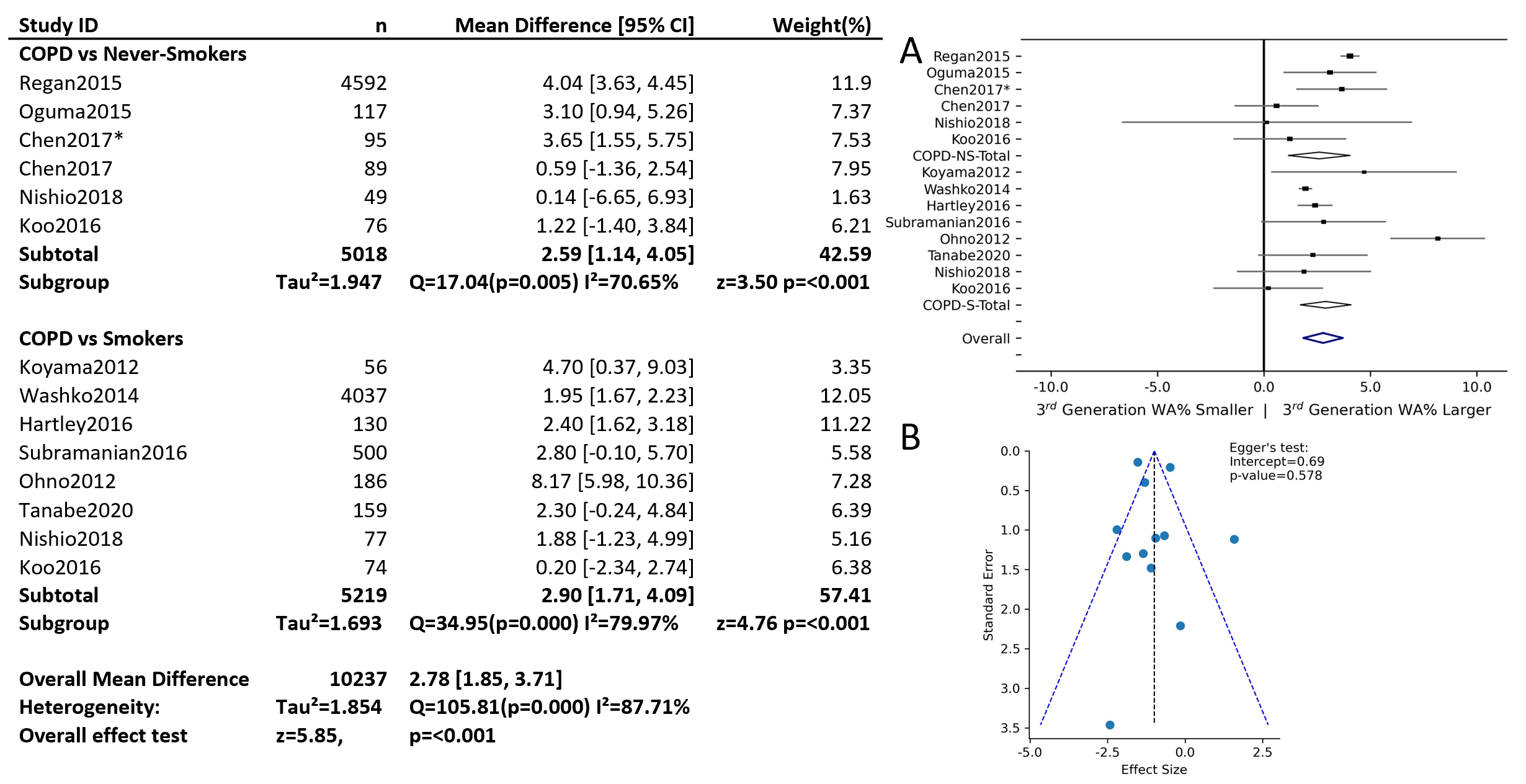

Supplement: Supplementary file 3 — Summary results for meta-analysis of COPD population 3rd generation Wall Area Percentage (WA%) vs controls. A) Forest plot of mean difference in WA% for individual studies. B) Forest plot of studies included in meta-analysis. COPD-NS-Total = COPD vs Never-Smokers mean difference. COPD-S-Total = COPD vs Smokers mean difference. Q = chi-squared. (PNG 304 kb) [file 330_2022_8600_MOESM3_ESM.png]
