# Supplementary material for: Bronchial wall parameters on CT in healthy never-smoking, smoking, COPD, and asthma populations: a systematic review and meta-analysis
Source: Eur Radiol. 2022 Feb 22;32(8):5308–18. doi: 10.1007/s00330-022-08600-1 (PMC9279249; doi:10.1007/s00330-022-08600-1)
Supplement: Supplementary file 4 — (DOCX 30 kb) [file 330_2022_8600_MOESM4_ESM.docx]

## Supplemental References

S1. Kirby M, Tanabe N, Tan WC, et al (2018) Total Airway Count on Computed Tomography and the Risk of Chronic Obstructive Pulmonary Disease Progression. Findings from a Population-based Study. American journal of respiratory and critical care medicine 197:56–65. https://doi.org/10.1164/rccm.201704-0692OC

S2. Koo H.-K., Hong Y., Lim M.N., et al (2016) Relationship between plasma matrix metalloproteinase levels, pulmonary function, bronchodilator response, and emphysema severity. International Journal of COPD 11:1129–1137. https://doi.org/10.2147/COPD.S103281

S3. Diaz AA, Hardin ME, Come CE, et al (2014) Childhood-onset asthma in smokers. association between CT measures of airway size, lung function, and chronic airflow obstruction. Annals of the American Thoracic Society 11:1371–1378. https://doi.org/10.1513/AnnalsATS.201403-095OC

S4. Diaz AA, Young TP, Maselli DJ, et al (2017) Bronchoarterial ratio in never-smokers adults: Implications for bronchial dilation definition. Respirology (Carlton, Vic) 22:108–113. https://doi.org/10.1111/resp.12875

S5. Halper-Stromberg E, Cho MH, Wilson C, et al (2017) Visual Assessment of Chest Computed Tomographic Images Is Independently Useful for Genetic Association Analysis in Studies of Chronic Obstructive Pulmonary Disease. Annals of the American Thoracic Society 14:33–40. https://doi.org/10.1513/AnnalsATS.201606-427OC

S6. Han MK, Kazerooni EA, Lynch DA, et al (2011) Chronic obstructive pulmonary disease exacerbations in the COPDGene study: associated radiologic phenotypes. Radiology 261:274–282. https://doi.org/10.1148/radiol.11110173

S7. Hardin M, Silverman EK, Barr RG, et al (2011) The clinical features of the overlap between COPD and asthma. Respiratory research 12:127–127. https://doi.org/10.1186/1465-9921-12-127

S8. Hardin M, Cho M, McDonald M-L, et al (2014) The clinical and genetic features of COPD-asthma overlap syndrome. The European respiratory journal 44:341–350. https://doi.org/10.1183/09031936.00216013

S9. Hersh CP, Zacharia S, Prakash Arivu Chelvan R, et al (2020) Immunoglobulin E as a Biomarker for the Overlap of Atopic Asthma and Chronic Obstructive Pulmonary Disease. Chronic obstructive pulmonary diseases (Miami, Fla) 7:1–12. https://doi.org/10.15326/jcopdf.7.1.2019.0138

S10. Kim SS, Seo JB, Lee HY, et al (2013) Chronic obstructive pulmonary disease: lobe-based visual assessment of volumetric CT by Using standard images--comparison with quantitative CT and pulmonary function test in the COPDGene study. Radiology 266:626–635. https://doi.org/10.1148/radiol.12120385

S11. Kim SS, Yagihashi K, Stinson DS, et al (2014) Visual Assessment of CT Findings in Smokers With Nonobstructed Spirometric Abnormalities in The COPDGene(®) Study. Chronic obstructive pulmonary diseases (Miami, Fla) 1:88–96. https://doi.org/10.15326/jcopdf.1.1.2013.0001#sthash.L0atdpjM.dpuf

S12. Kim V, Davey A, Comellas AP, et al (2014) Clinical and computed tomographic predictors of chronic bronchitis in COPD: a cross sectional analysis of the COPDGene study. Respiratory research 15:52–52. https://doi.org/10.1186/1465-9921-15-52

S13. Kim V., Desai P., Newell J.D., et al (2014) Airway wall thickness is increased in COPD patients with bronchodilator responsiveness. Respiratory Research 15:. https://doi.org/10.1186/s12931-014-0084-3

S14. Kinney GL, Santorico SA, Young KA, et al (2018) Identification of Chronic Obstructive Pulmonary Disease Axes That Predict All-Cause Mortality: The COPDGene Study. American journal of epidemiology 187:2109–2116. https://doi.org/10.1093/aje/kwy087

S15. Marchetti N, Garshick E, Kinney GL, et al (2014) Association between occupational exposure and lung function, respiratory symptoms, and high-resolution computed tomography imaging in COPDGene. American journal of respiratory and critical care medicine 190:756–762. https://doi.org/10.1164/rccm.201403-0493OC

S16. Martinez CH, Chen Y-H, Westgate PM, et al (2012) Relationship between quantitative CT metrics and health status and BODE in chronic obstructive pulmonary disease. Thorax 67:399–406. https://doi.org/10.1136/thoraxjnl-2011-201185

S17. Nambu A, Zach J, Schroeder J, et al (2016) Quantitative computed tomography measurements to evaluate airway disease in chronic obstructive pulmonary disease: Relationship to physiological measurements, clinical index and visual assessment of airway disease. European Journal of Radiology 85:2144–2151. https://doi.org/10.1016/j.ejrad.2016.09.010

S18. Regan EA, Lynch DA, Curran-Everett D, et al (2015) Clinical and Radiologic Disease in Smokers With Normal Spirometry. JAMA Intern Med 175:1539–1549. https://doi.org/10.1001/jamainternmed.2015.2735

S19. Schroeder JD, McKenzie AS, Zach JA, et al (2013) Relationships between airflow obstruction and quantitative CT measurements of emphysema, air trapping, and airways in subjects with and without chronic obstructive pulmonary disease. AJR American journal of roentgenology 201:W460–W470. https://doi.org/10.2214/AJR.12.10102

S20. Suh YJ, McDonald M-LN, Washko GR, et al (2018) Lung, Fat and Bone: Increased Adiponectin Associates with the Combination of Smoking-Related Lung Disease and Osteoporosis. Chronic obstructive pulmonary diseases (Miami, Fla) 5:134–143. https://doi.org/10.15326/jcopdf.5.2.2016.0174

S21. Sverzellati N, Lynch DA, Pistolesi M, et al (2014) PHYSIOLOGIC AND QUANTITATIVE COMPUTED TOMOGRAPHY DIFFERENCES BETWEEN CENTRILOBULAR AND PANLOBULAR EMPHYSEMA IN COPD. Chronic obstructive pulmonary diseases (Miami, Fla) 1:125–132. https://doi.org/10.15326/jcopdf.1.1.2014.0114

S22. Vazquez Guillamet R, Petersen H, Meek P, et al (2018) Grading Severity of Productive Cough Based on Symptoms and Airflow Obstruction. COPD 15:206–213. https://doi.org/10.1080/15412555.2018.1458218

S23. Vaz Fragoso CA, McAvay G, Van Ness PH, et al (2015) Phenotype of normal spirometry in an aging population. American journal of respiratory and critical care medicine 192:817–825. https://doi.org/10.1164/rccm.201503-0463OC

S24. Washko GR, Diaz AA, Kim V, et al (2014) Computed tomographic measures of airway morphology in smokers and never-smoking normals. Journal of Applied Physiology 116:668–673. https://doi.org/10.1152/japplphysiol.00004.2013

S25. Thomson NC, Chaudhuri R, Messow CM, et al (2013) Chronic cough and sputum production are associated with worse clinical outcomes in stable asthma. Respiratory medicine 107:1501–1508. https://doi.org/10.1016/j.rmed.2013.07.017

S26. Hong Y., Chae E.J., Seo J.B., et al (2012) Contributors of the severity of airflow limitation in COPD patients. Tuberculosis and Respiratory Diseases 72:8–14. https://doi.org/10.4046/trd.2012.72.1.8

S27. Koo HJ, Lee SM, Seo JB, et al (2019) Prediction of Pulmonary Function in Patients with Chronic Obstructive Pulmonary Disease: Correlation with Quantitative CT Parameters. Korean journal of radiology 20:683–692. https://doi.org/10.3348/kjr.2018.0391

S28. Lee JS, Huh JW, Chae EJ, et al (2012) Response patterns to bronchodilator and quantitative computed tomography in chronic obstructive pulmonary disease. Clinical physiology and functional imaging 32:12–18. https://doi.org/10.1111/j.1475-097X.2011.01046.x

S29. Lim JU, Lee J-H, Kim T-H, et al (2018) Alternative definitions of chronic bronchitis and their correlation with CT parameters. International journal of chronic obstructive pulmonary disease 13:1893–1899. https://doi.org/10.2147/COPD.S164055

S30. Park HJ, Lee SM, Choe J, et al (2019) Prediction of Treatment Response in Patients with Chronic Obstructive Pulmonary Disease by Determination of Airway Dimensions with Baseline Computed Tomography. Korean journal of radiology 20:304–312. https://doi.org/10.3348/kjr.2018.0204

S31. Han MK, Bartholmai B, Liu LX, et al (2009) Clinical significance of radiologic characterizations in COPD. COPD 6:459–467. https://doi.org/10.3109/15412550903341513

S32. Donohue KM, Hoffman EA, Baumhauer H, et al (2013) Asthma and lung structure on computed tomography: the Multi-Ethnic Study of Atherosclerosis Lung Study. The Journal of allergy and clinical immunology 131:361–8.e11. https://doi.org/10.1016/j.jaci.2012.11.036

S33. Smith BM, Hoffman EA, Basner RC, et al (2014) Not all measures of hyperinflation are created equal: lung structure and clinical correlates of gas trapping and hyperexpansion in COPD: the Multi-Ethnic Study of Atherosclerosis (MESA) COPD Study. Chest 145:1305–1315. https://doi.org/10.1378/chest.13-1884

S34. Dijkstra AE, Postma DS, ten Hacken N, et al (2013) Low-dose CT measurements of airway dimensions and emphysema associated with airflow limitation in heavy smokers: a cross sectional study. Respiratory research 14:11–11. https://doi.org/10.1186/1465-9921-14-11

S35. Mets OM, Schmidt M, Buckens CF, et al (2013) Diagnosis of chronic obstructive pulmonary disease in lung cancer screening Computed Tomography scans: independent contribution of emphysema, air trapping and bronchial wall thickening. Respiratory Research 14:59. https://doi.org/10.1186/1465-9921-14-59

S36. Mohamed Hoesein FAA, de Jong PA, Lammers J-WJ, et al (2013) Computed tomography structural lung changes in discordant airflow limitation. PloS one 8:e65177–e65177. https://doi.org/10.1371/journal.pone.0065177

S37. Mohamed Hoesein FAA, de Jong PA, Lammers J-WJ, et al (2014) Contribution of CT quantified emphysema, air trapping and airway wall thickness on pulmonary function in male smokers with and without COPD. COPD 11:503–509. https://doi.org/10.3109/15412555.2014.933952

S38. Hoesein FAAM, Jong PA de, Lammers J-WJ, et al (2015) Airway wall thickness associated with forced expiratory volume in 1 second decline and development of airflow limitation. European Respiratory Journal 45:644–651. https://doi.org/10.1183/09031936.00020714

S39. Pompe E, van Rikxoort EM, Mets OM, et al (2016) Follow-up of CT-derived airway wall thickness: Correcting for changes in inspiration level improves reliability. European journal of radiology 85:2008–2013. https://doi.org/10.1016/j.ejrad.2016.09.009

S40. Pompe E, de Jong PA, van Rikxoort EM, et al (2016) Smokers with emphysema and small airway disease on computed tomography have lower bone density. International journal of chronic obstructive pulmonary disease 11:1207–1216. https://doi.org/10.2147/COPD.S103680

S41. Takx RAP, Vliegenthart R, Mohamed Hoesein FAA, et al (2015) Pulmonary function and CT biomarkers as risk factors for cardiovascular events in male lung cancer screening participants: the NELSON study. European radiology 25:65–71. https://doi.org/10.1007/s00330-014-3384-6

S42. Xie X, Dijkstra AE, Vonk JM, et al (2014) Chronic respiratory symptoms associated with airway wall thickening measured by thin-slice low-dose CT. AJR American journal of roentgenology 203:W383–W390. https://doi.org/10.2214/AJR.13.11536

S43. Dransfield MT, Huang F, Nath H, et al (2010) CT emphysema predicts thoracic aortic calcification in smokers with and without COPD. COPD 7:404–410. https://doi.org/10.3109/15412555.2010.528085

S44. Yamashiro T, Matsuoka S, Estépar RSJ, et al (2010) Quantitative assessment of bronchial wall attenuation with thin-section CT: An indicator of airflow limitation in chronic obstructive pulmonary disease. AJR American journal of roentgenology 195:363–369. https://doi.org/10.2214/AJR.09.3653

S45. Aysola RS, Hoffman EA, Gierada D, et al (2008) Airway remodeling measured by multidetector CT is increased in severe asthma and correlates with pathology. Chest 134:1183–1191. https://doi.org/10.1378/chest.07-2779

S46. Choi S, Hoffman EA, Wenzel SE, et al (2017) Quantitative computed tomographic imaging-based clustering differentiates asthmatic subgroups with distinctive clinical phenotypes. The Journal of allergy and clinical immunology 140:690-700.e8. https://doi.org/10.1016/j.jaci.2016.11.053

S47. Shim SS, Schiebler ML, Evans MD, et al (2018) Lumen area change (Delta Lumen) between inspiratory and expiratory multidetector computed tomography as a measure of severe outcomes in asthmatic patients. The Journal of allergy and clinical immunology 142:1773-1780.e9. https://doi.org/10.1016/j.jaci.2017.12.1004

S48. Bhatt SP, Nath HP, Kim Y-I, et al (2018) Centrilobular emphysema and coronary artery calcification: mediation analysis in the SPIROMICS cohort. Respiratory research 19:257–257. https://doi.org/10.1186/s12931-018-0946-1

S49. Paulin LM, Smith BM, Koch A, et al (2018) Occupational Exposures and Computed Tomographic Imaging Characteristics in the SPIROMICS Cohort. Annals of the American Thoracic Society 15:1411–1419. https://doi.org/10.1513/AnnalsATS.201802-150OC

S50. Woodruff PG, Barr RG, Bleecker E, et al (2016) Clinical Significance of Symptoms in Smokers with Preserved Pulmonary Function. New England Journal of Medicine 374:1811–1821. https://doi.org/10.1056/NEJMoa1505971

S51. Zou C, Li F, Choi J, et al (2021) Longitudinal Imaging-Based Clusters in Former Smokers of the COPD Cohort Associate with Clinical Characteristics: The SubPopulations and Intermediate Outcome Measures in COPD Study (SPIROMICS). Int J Chron Obstruct Pulmon Dis 16:1477–1496. https://doi.org/10.2147/COPD.S301466

S52. Anazawa R, Kawata N, Matsuura Y, et al (2019) Longitudinal changes in structural lung abnormalities using MDCT in chronic obstructive pulmonary disease with asthma-like features. PloS one 14:e0227141–e0227141. https://doi.org/10.1371/journal.pone.0227141

S53. Bazan-Socha S, Jakiela B, Zuk J, et al (2021) Interactions via α2β1 cell integrin may protect against the progression of airway structural changes in asthma. International Journal of Molecular Sciences 22:. https://doi.org/10.3390/ijms22126315

S54. Brillet P-Y, Grenier PA, Fetita CI, et al (2013) Relationship between the airway wall area and asthma control score in moderate persistent asthma. European radiology 23:1594–1602. https://doi.org/10.1007/s00330-012-2743-4

S55. Boulet L-P, Boulay M-E, Coxson HO, et al (2021) Asthma with Irreversible Airway Obstruction in Smokers and Nonsmokers: Links between Airway Inflammation and Structural Changes. Respiration 99:1090–1100. https://doi.org/10.1159/000508163

S56. Camiciottoli G, Bigazzi F, Paoletti M, et al (2013) Pulmonary function and sputum characteristics predict computed tomography phenotype and severity of COPD. The European respiratory journal 42:626–635. https://doi.org/10.1183/09031936.00133112

S57. Capaldi DPI, Zha N, Guo F, et al (2016) Pulmonary Imaging Biomarkers of Gas Trapping and Emphysema in COPD: (3)He MR Imaging and CT Parametric Response Maps. Radiology 279:597–608. https://doi.org/10.1148/radiol.2015151484

S58. Chaudhuri R, McSharry C, Brady J, et al (2014) Low sputum MMP-9/TIMP ratio is associated with airway narrowing in smokers with asthma. The European respiratory journal 44:895–904. https://doi.org/10.1183/09031936.00047014

S59. Chauhan NS, Sood D, Takkar P, et al (2019) Quantitative assessment of airway and parenchymal components of chronic obstructive pulmonary disease using thin-section helical computed tomography. Polish journal of radiology 84:e54–e60. https://doi.org/10.5114/pjr.2019.82737

S60. Chae KJ, Jin GY, Choi J, et al (2021) Generation-based study of airway remodeling in smokers with normal-looking CT with normalization to control inter-subject variability. Eur J Radiol 138:109657. https://doi.org/10.1016/j.ejrad.2021.109657

S61. Chen H, Chen G-Q, Zeng Q-S, et al (2017) Quantitative Assessment of Airway Pathology in Subjects With COPD Using Low-Dose High-Resolution Computed Tomography. Respiratory care 62:953–962. https://doi.org/10.4187/respcare.05186

S62. Chen H, Zeng Q-S, Zhang M, et al (2017) Quantitative Low-Dose Computed Tomography of the Lung Parenchyma and Airways for the Differentiation between Chronic Obstructive Pulmonary Disease and Asthma Patients. Respiration; international review of thoracic diseases 94:366–374. https://doi.org/10.1159/000478531

S63. Choo JY, Lee KY, Shin C, et al (2014) Quantitative analysis of lungs and airways with CT in subjects with the chronic obstructive pulmonary disease (COPD) candidate FAM13A gene: case control study for CT quantification in COPD risk gene. Journal of computer assisted tomography 38:597–603. https://doi.org/10.1097/RCT.0000000000000077

S64. Crisafulli E, Alfieri V, Silva M, et al (2016) Relationships between emphysema and airways metrics at High-Resolution Computed Tomography (HRCT) and ventilatory response to exercise in mild to moderate COPD patients. Respiratory medicine 117:207–214. https://doi.org/10.1016/j.rmed.2016.06.016

S65. Dournes G, Laurent F, Coste F, et al (2015) Computed tomographic measurement of airway remodeling and emphysema in advanced chronic obstructive pulmonary disease. Correlation with pulmonary hypertension. American journal of respiratory and critical care medicine 191:63–70. https://doi.org/10.1164/rccm.201408-1423OC

S66. Gawlitza J, Haubenreisser H, Henzler T, et al (2018) Finding the right spot: Where to measure airway parameters in patients with COPD. European journal of radiology 104:87–93. https://doi.org/10.1016/j.ejrad.2018.05.003

S67. Gorska K, Korczynski P, Mierzejewski M, et al (2016) Comparison of endobronchial ultrasound and high resolution computed tomography as tools for airway wall imaging in asthma and chronic obstructive pulmonary disease. Respiratory medicine 117:131–138. https://doi.org/10.1016/j.rmed.2016.06.011

S68. Hao W, Li M, Pang Y, et al (2021) Increased chemokines levels in patients with chronic obstructive pulmonary disease: correlation with quantitative computed tomography metrics. Br J Radiol 94:20201030. https://doi.org/10.1259/bjr.20201030

S69. Hartley RA, Barker BL, Newby C, et al (2016) Relationship between lung function and quantitative computed tomographic parameters of airway remodeling, air trapping, and emphysema in patients with asthma and chronic obstructive pulmonary disease: A single-center study. The Journal of allergy and clinical immunology 137:1413-1422.e12. https://doi.org/10.1016/j.jaci.2016.02.001

S70. Hasegawa M, Nasuhara Y, Onodera Y, et al (2006) Airflow limitation and airway dimensions in chronic obstructive pulmonary disease. American journal of respiratory and critical care medicine 173:1309–1315. https://doi.org/10.1164/rccm.200601-037OC

S71. Hesselbacher SE, Ross R, Schabath MB, et al (2011) Cross-sectional analysis of the utility of pulmonary function tests in predicting emphysema in ever-smokers. International journal of environmental research and public health 8:1324–1340. https://doi.org/10.3390/ijerph8051324

S72. Hoshino M., Ohtawa J. (2013) Effects of tiotropium and salmeterol/fluticasone propionate on airway wall thickness in chronic obstructive pulmonary disease. Respiration 86:280–287. https://doi.org/10.1159/000351116

S73. Hoshino M, Ohtawa J (2014) Computed tomography assessment of airway dimensions with combined tiotropium and indacaterol therapy in COPD patients. Respirology (Carlton, Vic) 19:403–410. https://doi.org/10.1111/resp.12256

S74. Hoshino M, Ohtawa J, Akitsu K (2014) Increased C-reactive protein is associated with airway wall thickness in steroid-naive asthma. Annals of allergy, asthma & immunology : official publication of the American College of Allergy, Asthma, & Immunology 113:37–41. https://doi.org/10.1016/j.anai.2014.04.014

S75. Hoshino M, Ohtawa J, Akitsu K (2016) Effects of the addition of tiotropium on airway dimensions in symptomatic asthma. Allergy and asthma proceedings 37:147–153. https://doi.org/10.2500/aap.2016.37.3991

S76. Hoshino M, Ohtawa J, Akitsu K (2016) Effect of treatment with inhaled corticosteroid on serum periostin levels in asthma. Respirology (Carlton, Vic) 21:297–303. https://doi.org/10.1111/resp.12687

S77. Hoshino M, Ohtawa J, Akitsu K (2016) Association of airway wall thickness with serum periostin in steroid-naive asthma. Allergy and asthma proceedings 37:225–230. https://doi.org/10.2500/aap.2016.37.3945

S78. Hoshino M, Akitsu K, Kubota K (2019) Effect of Sublingual Immunotherapy on Airway Inflammation and Airway Wall Thickness in Allergic Asthma. The journal of allergy and clinical immunology In practice 7:2804–2811. https://doi.org/10.1016/j.jaip.2019.06.003

S79. Jiang D, Wang Z, Yu N, et al (2018) Airway Remodeling in Asthma: Evaluation in 5 Consecutive Bronchial Generations by Using High-Resolution Computed Tomography. Respiratory care 63:1399–1406. https://doi.org/10.4187/respcare.06050

S80. Jobst BJ, Weinheimer O, Buschulte T, et al (2019) Longitudinal airway remodeling in active and past smokers in a lung cancer screening population. European radiology 29:2968–2980. https://doi.org/10.1007/s00330-018-5890-4

S81. Karayama M, Inui N, Mori K, et al (2017) Respiratory impedance is correlated with morphological changes in the lungs on three-dimensional CT in patients with COPD. Scientific reports 7:41709–41709. https://doi.org/10.1038/srep41709

S82. Karayama M, Inui N, Mori K, et al (2018) Respiratory impedance is correlated with airway narrowing in asthma using three-dimensional computed tomography. Clinical and experimental allergy : journal of the British Society for Allergy and Clinical Immunology 48:278–287. https://doi.org/10.1111/cea.13083

S83. Karayama M, Inui N, Yasui H, et al (2019) Physiological and morphological differences of airways between COPD and asthma-COPD overlap. Scientific reports 9:7818–7818. https://doi.org/10.1038/s41598-019-44345-6

S84. Kim DJ, Kim C, Shin C, et al (2018) Impact of Model-Based Iterative Reconstruction on the Correlation between Computed Tomography Quantification of a Low Lung Attenuation Area and Airway Measurements and Pulmonary Function Test Results in Normal Subjects. Korean journal of radiology 19:1187–1195. https://doi.org/10.3348/kjr.2018.19.6.1187

S85. Kinose D, Ogawa E, Kawashima S, et al (2020) An index of the fractal characteristic of an airway tree is associated with airflow limitations and future body mass index reduction in COPD patients. Journal of Applied Physiology 128:1280–1286. https://doi.org/10.1152/japplphysiol.00461.2019

S86. Konietzke P, Wielpütz MO, Wagner WL, et al (2020) Quantitative CT detects progression in COPD patients with severe emphysema in a 3-month interval. European radiology 10.1007/s00330-019. https://doi.org/10.1007/s00330-019-06577-y

S87. Koyama H, Ohno Y, Nishio M, et al (2012) Three-dimensional airway lumen volumetry: comparison with bronchial wall area and parenchymal densitometry in assessment of airway obstruction in pulmonary emphysema. The British journal of radiology 85:1525–1532. https://doi.org/10.1259/bjr/22602417

S88. Kozlik P, Zuk J, Bartyzel S, et al (2020) The relationship of airway structural changes to blood and bronchoalveolar lavage biomarkers, and lung function abnormalities in asthma. Clinical and experimental allergy : journal of the British Society for Allergy and Clinical Immunology 50:15–28. https://doi.org/10.1111/cea.13501

S89. Kumar I, Verma A, Jain A, Agarwal SK (2018) Performance of quantitative CT parameters in assessment of disease severity in COPD: A prospective study. The Indian journal of radiology & imaging 28:99–106. https://doi.org/10.4103/ijri.IJRI_296_17

S90. Kurashima K, Hoshi T, Takayanagi N, et al (2012) Airway dimensions and pulmonary function in chronic obstructive pulmonary disease and bronchial asthma. Respirology 17:79–86. https://doi.org/10.1111/j.1440-1843.2011.02052.x

S91. Kurashima K, Hoshi T, Takaku Y, et al (2013) Changes in the airway lumen and surrounding parenchyma in chronic obstructive pulmonary disease. International journal of chronic obstructive pulmonary disease 8:523–532. https://doi.org/10.2147/COPD.S52637

S92. Lederlin M, Laurent F, Dromer C, et al (2012) Mean bronchial wall attenuation value in chronic obstructive pulmonary disease: comparison with standard bronchial parameters and correlation with function. AJR American journal of roentgenology 198:800–808. https://doi.org/10.2214/AJR.11.6895

S93. Mair G, Maclay J, Miller JJ, et al (2010) Airway dimensions in COPD: relationships with clinical variables. Respiratory medicine 104:1683–1690. https://doi.org/10.1016/j.rmed.2010.04.021

S94. Matsuo Y, Ogawa E, Seto-Yukimura R, et al (2019) Novel Respiratory Impedance-Based Phenotypes Reflect Different Pathophysiologies in Chronic Obstructive Pulmonary Disease Patients. International journal of chronic obstructive pulmonary disease 14:2971–2977. https://doi.org/10.2147/COPD.S224902

S95. Matsuoka S, Kurihara Y, Nakajima Y, et al (2005) Serial change in airway lumen and wall thickness at thin-section CT in asymptomatic subjects. Radiology 234:595–603. https://doi.org/10.1148/radiol.2342031466

S96. Matsuoka S, Kurihara Y, Yagihashi K, et al (2008) Airway dimensions at inspiratory and expiratory multisection CT in chronic obstructive pulmonary disease: correlation with airflow limitation. Radiology 248:1042–1049. https://doi.org/10.1148/radiol.2491071650

S97. Nishio M, Tanaka Y (2018) Heterogeneity in pulmonary emphysema: Analysis of CT attenuation using Gaussian mixture model. PloS one 13:e0192892–e0192892. https://doi.org/10.1371/journal.pone.0192892

S98. Niwa M, Fujisawa T, Karayama M, et al (2018) Differences in airway structural changes assessed by 3-dimensional computed tomography in asthma and asthma-chronic obstructive pulmonary disease overlap. Annals of allergy, asthma & immunology : official publication of the American College of Allergy, Asthma, & Immunology 121:704-710.e1. https://doi.org/10.1016/j.anai.2018.08.006

S99. Ohno Y, Koyama H, Yoshikawa T, et al (2012) Comparison of capability of dynamic O₂-enhanced MRI and quantitative thin-section MDCT to assess COPD in smokers. European journal of radiology 81:1068–1075. https://doi.org/10.1016/j.ejrad.2011.02.004

S100. Ostridge K, Williams NP, Kim V, et al (2018) Relationship of CT-quantified emphysema, small airways disease and bronchial wall dimensions with physiological, inflammatory and infective measures in COPD. Respiratory research 19:31–31. https://doi.org/10.1186/s12931-018-0734-y

S101. Patyk M, Obojski A, Sokołowska-Dąbek D, et al (2020) Airway wall thickness and airflow limitations in asthma assessed in quantitative computed tomography. Therapeutic advances in respiratory disease 14:1753466619898598–1753466619898598. https://doi.org/10.1177/1753466619898598

S102. Postma D.S., Brightling C., Baldi S., et al (2019) Exploring the relevance and extent of small airways dysfunction in asthma (ATLANTIS): baseline data from a prospective cohort study. The Lancet Respiratory Medicine 7:402–416. https://doi.org/10.1016/S2213-2600(19)30049-9

S103. Rice MB, Li W, Dorans KS, et al (2018) Exposure to Traffic Emissions and Fine Particulate Matter and Computed Tomography Measures of the Lung and Airways. Epidemiology (Cambridge, Mass) 29:333–341. https://doi.org/10.1097/EDE.0000000000000809

S104. Saure EW, Bakke PS, Lind Eagan TM, et al (2016) Diffusion capacity and CT measures of emphysema and airway wall thickness - relation to arterial oxygen tension in COPD patients. European clinical respiratory journal 3:29141–29141. https://doi.org/10.3402/ecrj.v3.29141

S105. Sayiner A, Hague C, Ajlan A, et al (2013) Bronchiolitis in young female smokers. Respiratory medicine 107:732–738. https://doi.org/10.1016/j.rmed.2012.12.023

S106. Shimizu K, Hasegawa M, Makita H, et al (2011) Comparison of airway remodelling assessed by computed tomography in asthma and COPD. Respiratory medicine 105:1275–1283. https://doi.org/10.1016/j.rmed.2011.04.007

S107. Suzuki T, Tada Y, Kawata N, et al (2015) Clinical, physiological, and radiological features of asthma-chronic obstructive pulmonary disease overlap syndrome. International journal of chronic obstructive pulmonary disease 10:947–954. https://doi.org/10.2147/COPD.S80022

S108. Tanabe N., Shima H., Sato S., et al (2019) Direct evaluation of peripheral airways using ultra-high-resolution CT in chronic obstructive pulmonary disease. European Journal of Radiology 120:. https://doi.org/10.1016/j.ejrad.2019.108687

S109. Tanabe N, Sato S, Oguma T, et al (2019) Associations of airway tree to lung volume ratio on computed tomography with lung function and symptoms in chronic obstructive pulmonary disease. Respiratory research 20:77–77. https://doi.org/10.1186/s12931-019-1047-5

S110. Tanabe N, Shimizu K, Terada K, et al (2021) Central airway and peripheral lung structures in airway disease-dominant COPD. ERJ Open Res 7:. https://doi.org/10.1183/23120541.00672-2020

S111. Telenga ED, Oudkerk M, van Ooijen PMA, et al (2017) Airway wall thickness on HRCT scans decreases with age and increases with smoking. BMC Pulm Med 17:. https://doi.org/10.1186/s12890-017-0363-0

S112. Tho NV, Trang LTH, Murakami Y, et al (2014) Airway wall area derived from 3-dimensional computed tomography analysis differs among lung lobes in male smokers. PloS one 9:e98335–e98335. https://doi.org/10.1371/journal.pone.0098335

S113. Tho NV, Ryujin Y, Ogawa E, et al (2015) Relative contributions of emphysema and airway remodelling to airflow limitation in COPD: Consistent results from two cohorts. Respirology (Carlton, Vic) 20:594–601. https://doi.org/10.1111/resp.12505

S114. Van Tho N, Ogawa E, Trang LTH, et al (2015) A mixed phenotype of airway wall thickening and emphysema is associated with dyspnea and hospitalization for chronic obstructive pulmonary disease. Annals of the American Thoracic Society 12:988–996. https://doi.org/10.1513/AnnalsATS.201411-501OC

S115. Wada Y, Kitaguchi Y, Yasuo M, et al (2018) Diversity of respiratory impedance based on quantitative computed tomography in patients with COPD. International journal of chronic obstructive pulmonary disease 13:1841–1849. https://doi.org/10.2147/COPD.S163129

S116. Wei X, Ding Q, Yu N, et al (2018) Imaging Features of Chronic Bronchitis with Preserved Ratio and Impaired Spirometry (PRISm). Lung 196:649–658. https://doi.org/10.1007/s00408-018-0162-2

S117. Wilson DO, Leader JK, Fuhrman CR, et al (2011) Quantitative computed tomography analysis, airflow obstruction, and lung cancer in the pittsburgh lung screening study. Journal of thoracic oncology : official publication of the International Association for the Study of Lung Cancer 6:1200–1205. https://doi.org/10.1097/JTO.0b013e318219aa93

S118. Xia T, Zheng W, Lure FYM, Guan Y (2021) CT phenotypes in mild-to-moderate chronic obstructive pulmonary disease: difference before and after the age of 60 years. Clin Radiol 76:273–280. https://doi.org/10.1016/j.crad.2020.11.112

S119. Yahaba M, Kawata N, Iesato K, et al (2014) The effects of emphysema on airway disease: correlations between multi-detector CT and pulmonary function tests in smokers. European journal of radiology 83:1022–1028. https://doi.org/10.1016/j.ejrad.2014.03.003

S120. Yang MS, Choi S, Choi Y, Jin KN (2018) Association Between Airway Parameters and Abdominal Fat Measured via Computed Tomography in Asthmatic Patients. Allergy, asthma & immunology research 10:503–515. https://doi.org/10.4168/aair.2018.10.5.503

S121. Yuan R, Hogg JC, Paré PD, et al (2009) Prediction of the rate of decline in FEV(1) in smokers using quantitative Computed Tomography. Thorax 64:944–949. https://doi.org/10.1136/thx.2008.112433

S122. Zhang Q, Illing R, Hui CK, et al (2012) Bacteria in sputum of stable severe asthma and increased airway wall thickness. Respiratory research 13:35–35. https://doi.org/10.1186/1465-9921-13-35

S123. Zhang X, Xia T, Lai Z, et al (2019) Uncontrolled asthma phenotypes defined from parameters using quantitative CT analysis. European radiology 29:2848–2858. https://doi.org/10.1007/s00330-018-5913-1

S124. Zhao Z, Jiang C, Zhao D, et al (2017) Two CHRN susceptibility variants for COPD are genetic determinants of emphysema and chest computed tomography manifestations in Chinese patients. International journal of chronic obstructive pulmonary disease 12:1447–1455. https://doi.org/10.2147/COPD.S134010
