# Supplementary material for: Bronchial wall parameters on CT in healthy never-smoking, smoking, COPD, and asthma populations: a systematic review and meta-analysis
Source: Eur Radiol. 2022 Feb 22;32(8):5308–18. doi: 10.1007/s00330-022-08600-1 (PMC9279249; doi:10.1007/s00330-022-08600-1)
Supplement: Supplementary file 5 — (DOCX 41 kb) [file 330_2022_8600_MOESM5_ESM.docx]

Table S1

| **Study** | **Database** | **Software** | **Wall Measuring Algorithm** | **Wall Measured from MPR Centreline** | **Parameters Normalised** |
| --- | --- | --- | --- | --- | --- |
| Kirby 2018 [S1] | CanCOLD | Apollo VIDA | Graph-Cut | Yes | No |
| Hong 2016 [26] | CODA | Unclear | Unclear | Unclear | No |
| Ji 2018 [16] | CODA | In-house | FWHM | Unclear | No |
| Koo 2016 [S2] | CODA | In-house | FWHM | Unclear | No |
| Bodduluri 2018 [58] | COPDGene | Pulmonary Workstation VIDA | Graph-Cut | Yes | No |
| Charbonnier 2019 [54] | COPDGene | Pulmonary Workstation VIDA | Graph-Cut | Yes | No |
| Diaz 2013 [24] | COPDGene | Pulmonary Workstation VIDA | Graph-Cut | Yes | No |
| Diaz 2014 [S3] | COPDGene | Pulmonary Workstation VIDA | Graph-Cut | Yes | No |
| Diaz 2017 [S4] | COPDGene | Pulmonary Workstation VIDA | Graph-Cut | Yes | No |
| Halper-Stromberg 2017 [S5] | COPDGene | Pulmonary Workstation VIDA | Graph-Cut | Yes | No |
| Han 2011 [S6] | COPDGene | Pulmonary Workstation VIDA | Graph-Cut | Yes | No |
| Hansel 2013 [49] | COPDGene | Pulmonary Workstation VIDA | Graph-Cut | Yes | No |
| Hardin 2011 [S7] | COPDGene | Pulmonary Workstation VIDA | Graph-Cut | Yes | No |
| Hardin 2014 [S8] | COPDGene | Pulmonary Workstation VIDA | Graph-Cut | Yes | No |
| Hersh 2020 [S9] | COPDGene | Pulmonary Workstation VIDA | Graph-Cut | Yes | No |
| Kim 2011 [28] | COPDGene | Pulmonary Workstation VIDA | Graph-Cut | Yes | No |
| Kim 2013 [S10] | COPDGene | Pulmonary Workstation VIDA | Graph-Cut | Yes | No |
| Kim 2014 [S11] | COPDGene | Pulmonary Workstation VIDA | Graph-Cut | Yes | No |
| Kim 2014 [S12] | COPDGene | Pulmonary Workstation VIDA | Graph-Cut | Yes | No |
| Kim 2014 [S13] | COPDGene | Pulmonary Workstation VIDA | Graph-Cut | Yes | No |
| Kinney 2018 [S14] | COPDGene | Pulmonary Workstation VIDA | Graph-Cut | Yes | No |
| Lowe 2019 [18] | COPDGene | Pulmonary Workstation VIDA | Graph-Cut | Yes | No |
| Machetti 2014 [S15] | COPDGene | Pulmonary Workstation VIDA | Graph-Cut | Yes | No |
| Martinez 2012 [S16] | COPDGene | Pulmonary Workstation VIDA | Graph-Cut | Yes | No |
| Nambu 2016 [S17] | COPDGene | Pulmonary Workstation VIDA | Graph-Cut | Yes | No |
| Regan 2015 [S18] | COPDGene | Pulmonary Workstation VIDA | Graph-Cut | Yes | No |
| Ross 2018 [7] | COPDGene | Pulmonary Workstation VIDA | Graph-Cut | Yes | No |
| Schroeder 2013 [S19] | COPDGene | Pulmonary Workstation VIDA | Graph-Cut | Yes | No |
| Suh 2018 [S20] | COPDGene | Pulmonary Workstation VIDA | Graph-Cut | Yes | No |
| Sverzellati 2014 [S21] | COPDGene | Pulmonary Workstation VIDA | Graph-Cut | Yes | No |
| Vasquez Guillamet 2018 [S22] | COPDGene | Pulmonary Workstation VIDA | Graph-Cut | Yes | No |
| VazFragoso 2015 [S23] | COPDGene | Pulmonary Workstation VIDA | Graph-Cut | Yes | No |
| Wan 2011 [50] | COPDGene | Pulmonary Workstation VIDA | Graph-Cut | Yes | No |
| Washko 2014 [S24] | COPDGene | Pulmonary Workstation VIDA | Graph-Cut | Yes | No |
| Zach 2012 [27] | COPDGene | Pulmonary Workstation VIDA | Graph-Cut | Yes | No |
| Subramanian  2016 [40] | EvA | Emphylx | FWHM | No | Yes |
| Thomson 2013 [S25] | Glasgow COPD and Asthma | In-house | FWHM | Yes | No |
| Thomson 2015 [14] | Glasgow COPD and Asthma | Pulmonary Workstation VIDA | Graph-Cut | Yes | No |
| Hong 2012 [S26] | KOLD | In-house | FWHM | Unclear | No |
| Koo 2019 [S27] | KOLD | AVIEW | Intensity-Integration | Yes | No |
| Lee 2012 [S28] | KOLD | In-house | FWHM | Unclear | No |
| Lim 2018 [S29] | KOLD | Unclear | FWHM | Unclear | No |
| Park 2019 [S30] | KOLD | AVIEW | Intensity-Integration | Yes | No |
| Diaz 2010 [19] | LTRC | Airway Inspector 3D Slicer | Phase Congruency | Yes | No |
| Han 2009 [S31] | LTRC | Unclear | FWHM | Yes | No |
| Donohue 2013 [S32] | MESA | In-house | FWHM | No | No |
| Smith 2014 [S33] | MESA | In-house | Unclear | Yes | No |
| Oelsner 2019 [8] | MESA, SPIROMICS | Apollo VIDA | Graph-Cut | Yes | No |
| Smith 2014 [59] | MESA, SPIROMICS | Apollo VIDA | Graph-Cut | Yes | No |
| Dijkstra 2013 [S34] | NELSON | MeVis | Intensity-Integration | Yes | No |
| Dijkstra 2015 [9] | NELSON | MeVis | Intensity-Integration | Yes | No |
| Mets 2013 [S35] | NELSON | MeVis | Intensity-Integration | Yes | No |
| Mohamed Hoesein 2013 [S36] | NELSON | MeVis | Intensity-Integration | Yes | No |
| Mohamed Hoesein 2014 [S37] | NELSON | CIRRUS Lung | Intensity-Integration | Yes | No |
| Mohamed Hoesein 2014 [21] | NELSON | CIRRUS Lung | Intensity-Integration | Yes | No |
| Mohamed Hoesein 2015 [S38] | NELSON | CIRRUS Lung | Intensity-Integration | Yes | No |
| Pompe 2016 [S39] | NELSON | CIRRUS Lung | Intensity-Integration | Yes | No |
| Pompe 2016 [S40] | NELSON | CIRRUS Lung | Intensity-Integration | Yes | No |
| Takx 2015 [S41] | NELSON | CIRRUS Lung | Intensity-Integration | Yes | No |
| Xie 2014 [S42] | NELSON | MeVis | Intensity-Integration | Yes | No |
| Dransfield 2010 [S43] | NLST | Airway Inspector 3D Slicer | FWHM | No | No |
| Gierada 2011 [15] | NLST | Airway Inspector 3D Slicer | FWHM | No | No |
| Lutey 2013 [47] | NLST | Airway Inspector 3D Slicer | Phase Congruency | No | No |
| Washko 2009 [45] | NLST | Airway Inspector 3D Slicer | FWHM | No | No |
| Yamashiro 2010 [S44] | NLST | Airway Inspector 3D Slicer | FWHM | No | No |
| Aysola 2008 [S45] | SARP | Pulmonary Workstation VIDA | Graph-Cut | Yes | Yes |
| Choi 2015 [37] | SARP | Pulmonary Workstation VIDA | Graph-Cut | Yes | Yes |
| Choi 2017 [S46] | SARP | Apollo VIDA | Graph-Cut | Yes | No |
| Shim 2018 [S47] | SARP | Pulmonary Workstation VIDA | Graph-Cut | Yes | Yes |
| Bhatt 2018 [S48] | SPIROMICS | Apollo VIDA | Graph-Cut | Yes | No |
| Haghighi 2018 [39] | SPIROMICS | Apollo VIDA | Graph-Cut | Yes | Yes |
| Paulin 2018 [S49] | SPIROMICS | Apollo VIDA | Graph-Cut | Yes | No |
| Woodruff 2016 [S50] | SPIROMICS | Apollo VIDA | Graph-Cut | Yes | No |
| Zou 2021 [S51] | SPIROMICS | Apollo VIDA | Graph-Cut |  |  |
| Cho 2019 [48] | SPIROMICS, SARP | Apollo VIDA | Graph-Cut | Yes | Yes |
| Choi 2017 [38] | SPIROMICS, SARP | Apollo VIDA | Graph-Cut | Yes | Yes |
| Anazawa 2019 [S52] |  | Airway Inspector 3D Slicer | FWHM | No | No |
| Bazan-Socha  2021 [S53] |  | AW Server | Unclear | Unclear | No |
| Brillet 2013 [S54] |  | BronCare | Unclear | Yes | Yes |
| Boulet 2021 [S55] |  | Apollo VIDA | Graph-Cut | Yes | No |
| Camiciottoli 2013 [S56] |  | Apollo VIDA | Graph-Cut | Yes | No |
| Capaldi 2016 [S57] |  | Pulmonary Workstation VIDA | Graph-Cut | Yes | No |
| Chaudhuri 2014 [S58] |  | Pulmonary Workstation VIDA | Graph-Cut | Yes | No |
| Chauhan 2019 [S59] |  | In-house | Manual | Yes | No |
| Chae 2021 [S60] |  | Apollo VIDA | Graph-Cut | Yes | No |
| Chen 2017 [S61] |  | Apollo VIDA | Graph-Cut | Yes | No |
| Chen 2017 [S62] |  | Apollo VIDA | Graph-Cut | Yes | Yes |
| Choo 2014 [S63] |  | AVIEW | FWHM | Unclear | No |
| Crisafulli 2016 [S64] |  | MeVis | Intensity-Integration | Unclear | No |
| Dournes 2015 [S65] |  | In-house | Laplacian-of-Gaussian | Yes | No |
| Eddy 2020 [55] |  | Pulmonary Workstation VIDA | Graph-Cut | Yes | No |
| Gawlitza 2018 [S66] |  | Syngo.Pulmo3D | Unclear | Yes | No |
| Gietema 2013 [25] |  | Pulmonary Workstation VIDA | Graph-Cut | Yes | No |
| Gorska 2016 [S67] |  | Kroton Measurements | Manual | No | No |
| Gupta 2014 [44] |  | Pulmonary Workstation VIDA | Graph-Cut | Yes | Yes |
| Hackx 2017 [23] |  | Siemens Virtual Bronchoscopy | FWHM | Yes | No |
| Hao 2021 [S68] |  | COPD Analysis Phillips | Unclear | Yes | No |
| Hartley 2016 [S69] |  | Apollo VIDA | Graph-Cut | Yes | Yes |
| Hasegawa 2006 [S70] |  | AZE | FWHM | Yes | No |
| Hesselbacher 2011 [S71] |  | EmphylxJ | FWHM | No | No |
| Higami 2016 [20] |  | Apollo VIDA | Graph-Cut | Yes | No |
| Hoshino 2012 [10] |  | Unclear | FWHM | No | Yes |
| Hoshino 2013 [S72] |  | Unclear | FWHM | No | Yes |
| Hoshino 2014 [S73] |  | Unclear | FWHM | No | Yes |
| Hoshino 2014 [S74] |  | Unclear | FWHM | No | Yes |
| Hoshino 2016 [S75] |  | Unclear | FWHM | Unclear | Yes |
| Hoshino 2016 [S76] |  | Unclear | FWHM | No | Yes |
| Hoshino 2016 [S77] |  | Unclear | FWHM | No | Yes |
| Hoshino 2019 [S78] |  | Unclear | FWHM | No | Yes |
| Inoue 2014 [51] |  | Unclear | Manual | Unclear | Yes |
| Jiang 2018 [S79] |  | Unclear | Unclear | Yes | No |
| Jobst 2019 [S80] |  | YACTA | Intensity-Integration | Yes | No |
| Kambara 2014 [43] |  | AZE | FWHM | Yes | No |
| Karayama 2017 [S81] |  | Synapse Vincent | Graph-Cut | Yes | No |
| Karayama 2018 [S82] |  | Synapse Vincent | Graph-Cut | Yes | No |
| Karayama 2019 [S83] |  | Synapse Vincent | Graph-Cut | Yes | Yes |
| Kim 2017 [52] |  | Pulmonary Workstation VIDA | Graph-Cut | Yes | No |
| Kim 2018 [S84] |  | IntelliSpace | Weinheimer | Unclear | No |
| Kinose 2020 [S85] |  | Pulmonary Workstation VIDA | Graph-Cut | Yes | No |
| Kirby 2015 [17] |  | Pulmonary Workstation VIDA | Graph-Cut | Unclear | Yes |
| Konietzke 2020 [S86] |  | YACTA | Intensity-Integration | Unclear | Yes |
| Koyama 2012 [S87] |  | Matlab | FWHM | Unclear | No |
| Koyama 2014 [41] |  | Matlab | FWHM | No | No |
| Kozlik 2020 [S88] |  | AW Server Software | Unclear | Yes | No |
| Kumar 2018 [S89] |  | Pulmonary Workstation VIDA | Graph-Cut | Yes | No |
| Kurashima 2012 [S90] |  | Philips Custom | FWHM | Yes | No |
| Kurashima 2013 [S91] |  | Unclear | Unclear | Yes | No |
| Lederlin 2012 [S92] |  | Myrian | Manual | No | No |
| Li 2018 [53] |  | FACT-Digital | Unclear | Yes | No |
| Li 2018 [11] |  | FACT-Digital Lung | Principal Curvature | Unclear | No |
| Liu 2015 [13] |  | Lung Enhanced | Manual | No | Yes |
| Mair 2010 [S93] |  | In-house | FWHM | Yes | No |
| Matsuo 2019 [S94] |  | Apollo VIDA | Graph-Cut | Yes | No |
| Matsuoka 2005 [S95] |  | Unclear | Thresholding | No | No |
| Matsuoka 2008 [S96] |  | Ziostation | FWHM | Yes | No |
| Nakano 2000 [36] |  | In-house | FWHM | No | Yes |
| Nishio 2018 [S97] |  | Airway Inspector 3D Slicer | FWHM | No | No |
| Niwa 2018 [S98] |  | Synapse Vincent | Graph-Cut | Unclear | Yes |
| Oguma 2015 [56] |  | In-house | FWHM | Yes | Yes |
| Ohno 2012 [S99] |  | AZE | Unclear | Yes | No |
| Ostridge 2018 [S100] |  | Apollo VIDA | Graph-Cut | Yes | No |
| Patyk 2020 [S101] |  | Syngo.Pulmo3D | FWHM | Yes | No |
| Petersen 2014 [42] |  | In-house | Graph-Cut | Yes | No |
| Postma 2019 [S102] |  | Apollo VIDA | Graph-Cut | Yes | Yes |
| Rice 2018 [S103] |  | Airway Inspector 3D Slicer | FWHM | No | No |
| Saure 2016 [S104] |  | Pulmonary Workstation VIDA | Graph-Cut | Yes | No |
| Sayiner 2013 [S105] |  | EmphylxJ | FWHM | No | No |
| Shimizu 2010 [S106] |  | AZE | FWHM | Yes | Yes |
| Shimizu 2011 [4] |  | Unclear | FWHM | Yes | No |
| Suzuki 2015 [S107] |  | Aze, AZE Ltd | FWHM | Yes | No |
| Takayanagi 2017 [12] |  | Airway Inspector 3D Slicer | FWHM | No | No |
| Tanabe 2019 [S108] |  | In-house | FWHM | Yes | No |
| Tanabe 2019 [57] |  | Synapse Vincent | Graph-Cut | Yes | No |
| Tanabe 2020 [S109] |  | In-house | FWHM | Yes | No |
| Tanabe 2021 [S110] |  | In-house | FWHM | Yes | Yes |
| Telenga 2017 [S111] |  | MeVis | Intensity-Integration | Yes | No |
| Tho 2014 [S112] |  | Pulmonary Workstation VIDA | Graph-Cut | Yes | No |
| Tho 2015 [S113] |  | Apollo VIDA | Graph-Cut | Yes | No |
| VanTho 2015 [S114] |  | Apollo VIDA | Graph-Cut | Yes | No |
| Wada 2018 [S115] |  | ZioCube | Unclear | Yes | No |
| Wei 2018 [S116] |  | FACT-Digital Lung | Principal Curvature | Yes | No |
| Wilson 2011 [S117] |  | In-house | Partial Membership | Unclear | No |
| Xia 2021 [S118] |  | Apollo VIDA | Graph-Cut | Yes | Yes |
| Yahaba 2014 [S119] |  | Ziostation 2 | FWHM | Yes | No |
| Yang 2018 [S120] |  | Unclear | Unclear | Unclear | Yes |
| Yuan 2009 [S121] |  | EmphylxJ | FWHM | No | No |
| Zhang 2012 [S122] |  | Unclear | Manual | No | No |
| Zhang 2019 [S123] |  | Pulmonary Workstation VIDA | Graph-Cut | Yes | Yes |
| Zhao 2017 [S124] |  | Apollo VIDA | Graph-Cut | Yes | No |

Summary characteristics of articles included in the systematic review. MPR: Multiplanar Reconstruction, FWHM: Full-Width Half-Maximum.
