# Supplementary material for: Bronchial wall parameters on CT in healthy never-smoking, smoking, COPD, and asthma populations: a systematic review and meta-analysis
Source: Eur Radiol. 2022 Feb 22;32(8):5308–18. doi: 10.1007/s00330-022-08600-1 (PMC9279249; doi:10.1007/s00330-022-08600-1)
Supplement: Supplementary file 6 — (DOCX 30 kb) [file 330_2022_8600_MOESM6_ESM.docx]

Table S2

| **Study** | **Database** | **Software** | **Bronchial Measurements Normalised?** | **Sequence Generation** | **Allocation Concealment** | **Incomplete Outcome Data** | **Selective Outcome Reporting** | **Other Sources of Bias** |
| --- | --- | --- | --- | --- | --- | --- | --- | --- |
| **Cho 2019 [48]** | SPIROMICS | Apollo VIDA | No | Low | Low | Low | Low | Low |
| **Paulin 2018 [S49]** | SPIROMICS | Apollo VIDA | No | Low | Low | Low | Low | Low |
| **Woodruff 2016 [S50]** | SPIROMICS | Apollo VIDA | No | Low | Low | Low | Low | Low |
| **Aysola 2008 [S45]** | SARP | Pulmonary Workstation VIDA | No | Low | Low | Low | Low | Low |
| **Gierada 2011 [15]** | NLST | Airway Inspector 3D Slicer | No | Low | Low | Low | Low | Low |
| **Lutey 2013 [47]** | NLST | Airway Inspector 3D Slicer | No | Low | Low | Low | Low | Low |
| **Mohamed Hoesein 2013 [S36]** | NELSON | MeVis | No | Low | Low | Low | Low | Male smokers, Dutch |
| **Diaz 2010 [19]** | LTRC | Airway Inspector 3D Slicer | No | Low | Low | Low | Low | Low |
| **Han 2009 [S31]** | LTRC | Unclear | No | Low | Low | Low | Low | Low |
| **Hong 2012 [S26]** | KOLD | In-House | No | Low | Low | Low | Low | Male Korean. Unclear methods for WAP |
| **Koo 2019 [S27]** | KOLD | AVIEW | No | Low | Low | No breakdown of WA%, Ai and WT values | Low | Predominantly male korean population. |
| **Lee 2012 [S28]** | KOLD | In-House | No | Low | Low | Low | Low | Pred male korean. |
| **Lim 2018 [S29]** | KOLD | Unclear | No | Low | Low | Low | Low | Majority male, Korean |
| **Park 2019 [S30]** | KOLD | AVIEW | No | Low | Low | Low | Low | Predominantly male korean copd population on treatment. |
| **Charbonnier 2019 [54]** | COPDGene | Pulmonary Workstation VIDA | No | Low | Low | Low | Low | Low |
| **Halper-Stromberg 2017 [S5]** | COPDGene | Pulmonary Workstation VIDA | No | Low | Low | Low | Low | Low |
| **Regan 2015 [S18]** | COPDGene | Pulmonary Workstation VIDA | No | Low | Low | Low | Low | Low |
| **Washko 2014 [S24]** | COPDGene | Pulmonary Workstation VIDA | No | Low | Low | Low | Low | Low |
| **Anazawa 2019 [S52]** |  | Airway Inspector 3D Slicer | No | Low | Low | Low | Low | Low |
| **Boulet 2021 [S55]** |  | Apollo VIDA | No | Low | Low | Low | Low | Low |
| **Brillet 2013 [S54]** |  | BronCare | BSA | Low | Low | Low | Low | Low |
| **Camiciottoli 2013 [S56]** |  | Apollo VIDA | No | Low | Unclear how the 100 patients for the learning set were selected. | Low | Low | Low |
| **Chauhan 2019 [S59]** |  | In-House | No | Unclear Inclusion/Exclusion criteria | Low | Low | Low | Manual measurements. |
| **Chen 2017 [S61]** |  | Apollo VIDA | BSA | Low | Low | Low | Low | Low |
| **Chen 2017 2 [S62]** |  | Apollo VIDA | No | Low | Low | Low | Low | Low |
| **Dournes 2015 [S65]** |  | In-House | No | Low | Low | Low | Low | Contains patients with pulmonary hypertension. |
| **Eddy 2020 [55]** |  | Pulmonary Workstation VIDA | No | Low | Low | Low | Low | Low |
| **Gietema 2013 [25]** |  | Pulmonary Workstation VIDA | No | Low | Low | Low | Low | Low |
| **Gupta 2014 [44]** |  | Pulmonary Workstation VIDA | BSA | Low | Low | Low | Some Concern | Low |
| **Hackx 2017 [23]** |  | Siemens Virtual Bronchoscopy | No | Low | Low | Low | Low | Low |
| **Hao 2021 [S68]** |  | COPD Analysis Phillips | No | Low | Low | Low | Low | Low |
| **Hartley 2016 [S69]** |  | Apollo VIDA | BSA | Low | Low | Low | Low | Low |
| **Hasegawa 2006**  **[S70]** |  | AZE | No | Low | Low | Low | Low | Majority Male participants. |
| **Higami 2016 [20]** |  | Apollo VIDA | No | Low | Low | Low | Low | 92% Male population. |
| **Jiang 2018 [S79]** |  | Unclear | No | Low | Low | Some Concern | Missing RB1 values for LA, WA | Unclear Segmentation and Measurement Methods |
| **Kim 2017 [52]** |  | Pulmonary Workstation VIDA | No | Low | Low | Unclear what generations and airways included | Low | Korean only cohort |
| **Kim 2018 [S84]** |  | IntelliSpace | No | Low | Low | Low | Low | Korean elderly smokers |
| **Kirby 2015 [17]** |  | Pulmonary Workstation VIDA | No | Low | Low | Low | Low | Low |
| **Kirby 2018 [S1]** | CanCOLD | Apollo VIDA | No | Low | Low | Low | Low | Low |
| **Koo 2016 [S2]** | CODA | In-House | No | Low | Low | Low | Low | Participants living near cement plants. |
| **Koyama 2012 [S87]** |  | Matlab In-House | No | Low | Low | Low | Low | Low |
| **Lederlin 2012 [S92]** |  | Myrian | No | Low | Low | Unclear SD for WA% | Low | Low |
| **Li 2018 [53]** |  | FACT-Digital | No | Low | Low | Some Concern | Low | All male. No indication of COPD status. Cluster of participants from one region. |
| **Mair 2010 [S93]** |  | In-House | No | Low | Low | Low | Low | Low |
| **Matsuoka 2005 [S95]** |  | Unclear | No | Low | Low | Low | Low | Low |
| **Matsuoka 2008 [S96]** |  | Unclear | No | Retrospective Study | Low | Low | Low | Low |
| **Nakano 2000 [36]** |  | In-House | BSA | Low | Low | Low | Low | Low |
| **Nishio 2018 [S97]** |  | Airway Inspector 3D Slicer | No | Low | Low | Low | Low | Retrospective study including smokers that have presented for other reasons |
| **Oguma 2015 [56]** |  | In-House | BSA | Low | Low | Low | Low | Low |
| **Ohno 2012 [S99]** |  | AZE | No | Low | Low | Low | Low | Low |
| **Ostridge 2018 [S100]** |  | Apollo VIDA | No | Low | Low | Low | Low | Low |
| **Saure 2016 [S104]** |  | Pulmonary Workstation VIDA | No | Low | Low | Low | Low | Low |
| **Sayiner 2013 [S105]** |  | EmphylxJ | No | Low | Low | Low | Low | Female, young, high education |
| **Subramanian 2016 [40]** |  | Emphylx | BSA | Low | Low | Low | Low | Low |
| **Takayanagi 2017 [12]** |  | Airway Inspector 3D Slicer | No | Low | Low | Low | Low | Majority Male participants. |
| **Tanabe 2019 [S108]** |  | In-House | No | Low | Low | Low | Low | Male only |
| **Tanabe 2020 [109]** |  | In-House | No | Low | Low | Low | Low | Low |
| **Tanabe 2021 [110]** |  | In-House | BSA | Low | Low | Low | Low | Low |
| **Tho 2015 [S113]** |  | Apollo VIDA | No | Low | Low | Low | Low | Male only |
| **Van Tho 2015 [S114]** |  | Apollo VIDA | No | Low | Low | Low | Low | Male only chinese population. |
| **Wada 2018 [S115]** |  | ZioCube | No | Low | Low | No overall statistics for subjects | Low | Low |
| **Xia 2021 [S118]** |  | Apollo VIDA | BSA | Low | Low | Low | Low | Low |
| **Yahaba 2014 [S119]** |  | Ziostation 2 | No | Low | High | Low | Low | Unclear number of male/female participants. |
| **Yuan 2009 [S121]** |  | EmphylxJ | No | Low | Low | Low | Low | No breakdown between Smokers and COPD |
| **Zhang 2012 [S122]** |  | Unclear | No | Low | Low | Low | Low | Low |
| **Zhang 2019 [S123]** |  | Pulmonary Workstation VIDA | BSA | Low | Low | Low | Low | Low |

Risk of Bias Assessment for studies included in the pooling of bronchial parameters.
